# Extracellular Vesicles in Veterinary Medicine

**DOI:** 10.3390/ani12192716

**Published:** 2022-10-10

**Authors:** Valentina Moccia, Alessandro Sammarco, Laura Cavicchioli, Massimo Castagnaro, Laura Bongiovanni, Valentina Zappulli

**Affiliations:** 1 Department of Comparative Biomedicine and Food Science, University of Padova, 35020 Legnaro, Italy; 2 Department of Microbiology, Immunology and Molecular Genetics, University of California, Los Angeles, CA 90095, USA; 3 Faculty of Veterinary Medicine, University of Teramo, 64100 Teramo, Italy; 4 Department of Biomolecular Health Sciences, Faculty of Veterinary Medicine, Utrecht University, 3584 Utrecht, The Netherlands

**Keywords:** veterinary pathology, veterinary physiology, biomarkers, therapy

## Abstract

**Simple Summary:**

Extracellular vesicles (EVs) are small vesicles released by human and animal cells, parasites, microorganisms, and plants. They travel within bodily fluids, transferring the content of their cell of origin to other cells, being both intra- and inter-organism messengers. This EV-mediated method of communication governs many normal functions as well as disease processes. Because of this important role, EVs have been largely studied since 1984, mainly in humans, but more recently also in animals, parasites, and bacteria. In this review, we explore the literature on EVs in animals between 1984 and 2021 and summarize the most important results of approximately 220 scientific papers. Results are presented based on the main topic of research, such as EVs in physiology and pathophysiology, use of EVs as markers to diagnose diseases, or as possible natural transporters of therapies or vaccines. Since working with EVs is challenging, we also address the critical technical points found in the veterinary literature. Finally, we included a brief summary on EVs shed within animal milk, an area of large interest for the multiple applications for human health.

**Abstract:**

Extracellular vesicles (EVs) are cell-derived membrane-bound vesicles involved in many physiological and pathological processes not only in humans but also in all the organisms of the eukaryotic and prokaryotic kingdoms. EV shedding constitutes a fundamental universal mechanism of intra-kingdom and inter-kingdom intercellular communication. A tremendous increase of interest in EVs has therefore grown in the last decades, mainly in humans, but progressively also in animals, parasites, and bacteria. With the present review, we aim to summarize the current status of the EV research on domestic and wild animals, analyzing the content of scientific literature, including approximately 220 papers published between 1984 and 2021. Critical aspects evidenced through the veterinarian EV literature are discussed. Then, specific subsections describe details regarding EVs in physiology and pathophysiology, as biomarkers, and in therapy and vaccines. Further, the wide area of research related to animal milk-derived EVs is also presented in brief. The numerous studies on EVs related to parasites and parasitic diseases are excluded, deserving further specific attention. The literature shows that EVs are becoming increasingly addressed in veterinary studies and standardization in protocols and procedures is mandatory, as in human research, to maximize the knowledge and the possibility to exploit these naturally produced nanoparticles.

## 1. Introduction

Extracellular vesicles (EVs) are heterogenous membrane-bound vesicles released by cells and involved in intercellular communication. Many EV-subtypes are now recognized, classified mainly for their biogenesis or for their size [1]. The outward budding of the plasma membrane produces ectosomes (microvesicles/microparticles), while the fusion of the plasma membrane with multivesicular bodies, derived from endosomes, originates exosomes [2]. However, since to date biogenesis can only be confirmed through live imaging techniques, EVs are mainly classified according to their size in small EVs (<200 nm) or medium-large EVs (>200 nm) [1]. More recently new EV-subtypes have also been described, such as large (1–10 µm) tumor-derived EVs named oncosomes [3].

EVs are a preserved evolutionary mechanism of communication that mediate the crosstalk between cells within a body, but also between different organisms of the same or even different species [4]. After the release from a donor cell, locally or at distance travelling through body fluids, EVs can interact with a recipient cell through not fully understood mechanisms, including direct contact through ligand–receptor interaction, fusion of EV membrane with the plasma cell membrane and, more often, endocytosis [2,5,6]. EV cargo is complex, made of proteins, lipids, DNA, and small non-coding RNA [2,5]. This complex cargo is responsible for most of the very wide and heterogenous effects of EVs in normal physiological processes as well as in pathological progression [2,5]. Moreover, EVs also have a discarding function, since they often target lysosomes leading to the elimination of molecules (e. g. proteins, lipids) from cells and to the creation of new metabolites [7].

**Figure 1:**
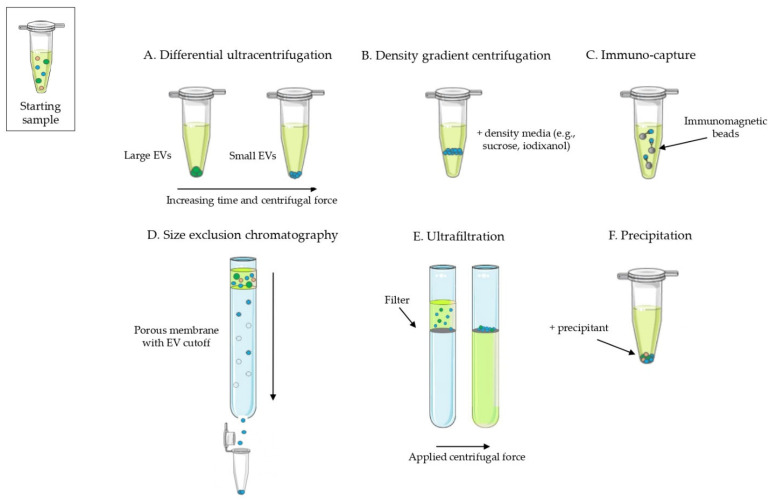
Schematic representation of the most common methods to isolate extracellular vesicles. (**A**) Separation of EVs with differential ultracentrifugation; larger EVs are isolated with lower *g* forces compared to small EVs. (**B**) Separation of EVs according to their density in density gradient centrifugation. (**C**) EVs are capture using specific antibodies against exposed antigens; here immunomagnetic beads bear the antibodies but also plate and chip systems are available. (**D**) Separation of EVs through size exclusion chromatography; while smaller particles are trapped in the porous matrix, larger particles and EVs elute earlier from the column. (**E**) Ultrafiltration allows the concentration of particles larger than the cutoff size of the filter. (**F**) In precipitation, a precipitating agent causes sedimentation of EVs and other particles.

EVs can be isolated from body fluids, cell culture media and from tissues with many techniques (Figure 1) [1]. Differential ultracentrifugation, which exploits increasing centrifugal forces to pellet EVs, has been the gold standard for years, but nowadays, different techniques can be applied and combined to reach the required recovery rate or to purify a specific EV subtype [1,8]. Since different EV isolation methods result in different EV quantities and subtypes, EV presence should always be assessed after purification, characterizing EVs for quantity (e. g. number of particles or protein/lipid content) and for the presence of EV markers and of other co-isolated components [1].

EVs are now investigated for wide potential effects in many research fields, but their study first started in human medicine during the mid of the XX century, when the presence of pelleted particles in high-speed centrifuged blood was first described [9]. During the 1960s and the 1970s, many electron microscopy (EM) studies described the release of vesicle-like structures by cells, but the real starting point of EV research can be dated in 1983, thanks to two publications of the Johnstone and Stahl laboratories [10,11]. These two papers aimed to follow the fate of the transferrin receptor and described through EM the presence of multivesicular bodies in reticulocytes and the release of vesicles upon fusion with the plasma membrane [9,10,11]. From that moment on, the studies investigating the role and the composition of these vesicles increased exponentially, leading to the tremendous increase of interest during the last two decades. Progressively, since the beginning of the XXI Century, the interest in EVs has grown in many other research areas, including veterinary medicine (VM). With the growing of EV-specific studies and publications on disparate areas of research, it became clear that EVs, with the ability to mediate intercellular transfer functions, play a key role in the physiology and pathophysiology not only in humans but also in other organisms, such as plants, animals, bacteria, and virus, with emerging evidence of EV importance also in inter-organisms’ communication [12,13]. A preserved process of EV formation and release across a broad range of species has been demonstrated, strongly suggesting the essential function of EVs in all forms of life [14].

**Figure 2:**
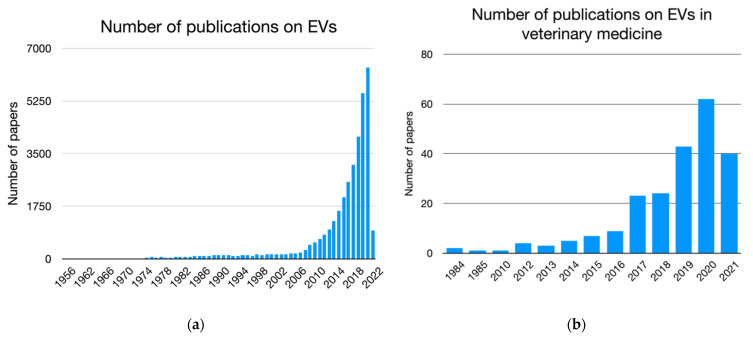
(**a**) Bar graph representing the number of papers on extracellular vesicles (EVs) in the literature; (**b**) bar graph representing the number of papers on EVs in veterinary medicine literature.

In VM, EV research is a relatively new, but rapidly expanding, field of research, and published research studies on EVs are increasing every year, investigating different roles of EVs in animal physiology, pathology, and their potential applications as diagnostic biomarkers and in therapy. Figure 2a,b show how, despite the delayed and much smaller number of papers, the exponential growth of scientific publications on EVs in VM is similar to the whole EV-related literature (Figure 1a,b). The increase of EV studies in VM mainly reflects worldwide contemporary concerns in the concept of “one health” such as the need for high productivity and welfare in food animal husbandry, control of emerging infectious disease and zoonosis and environmental monitoring [15,16,17]. Understanding how EVs can act in physiology and pathology of animals could be of great importance in the whole picture of EV research. Moreover, animals and humans show similar physiology and share several diseases, such as cancer, making animals also good spontaneous and inducible models for human research [18,19].

With the present review paper, the authors intend to summarize the current status of the EV research performed on domestic and wild animals, analyzing the content of scientific literature, including approximately 220 papers published since 1984. Studies on EVs related to parasites and parasitic diseases are excluded. The literature search was conducted on Pubmed database filtering for “other animals” and applying as major keywords: extracellular vesicles, exosomes, microvesicles, and oncosomes. Search within the literature was limited to November 2021.

**Figure 3:**
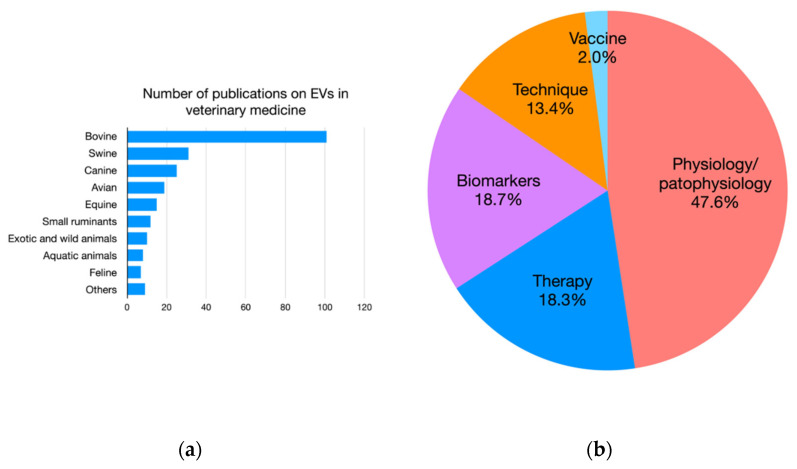
(**a**) Bar graph representing the number of papers on EVs by non-human species. (**b**) Pie chart representing the percentage of papers on EVs by application.

Most of the EV-related studies in VM have been conducted on pets and farm animals, especially on bovines, pigs, and dogs, but interestingly aquatic organisms and exotic and wild animals are also considered in preliminary EV studies (Figure 3a). The main focus of these studies is related to understanding the role of EVs in physiology and pathophysiology, with fewer but promising works related to the application of EVs as biomarkers or in therapy (Figure 3b). More recently, applications in wild/aquatic animals for environmental monitoring have appeared. Furthermore, a very broad area of interest of EV research in VM regards milk-derived EVs for their role as loadable and natural carriers with therapeutic potentials and as mediators of inter-organisms communication [20].

**Figure 4:**
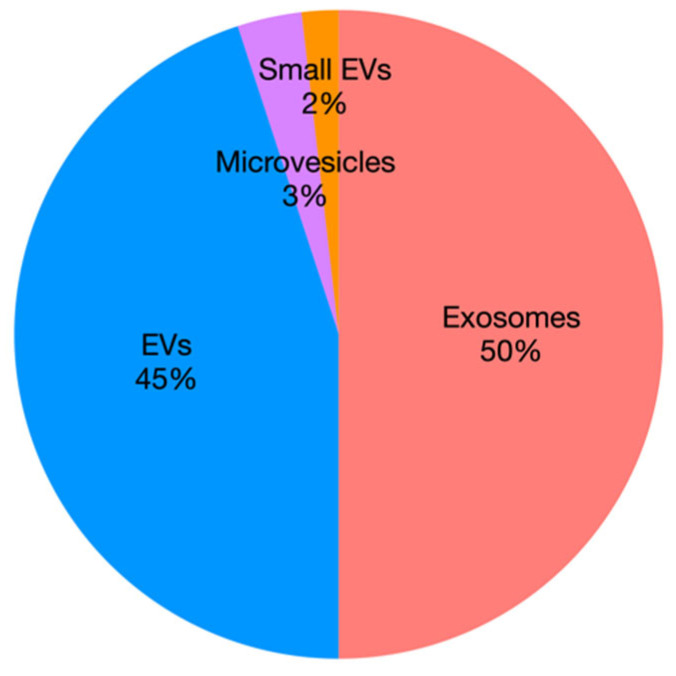
Pie chart representing the percentage of papers by EV subtype.

Small EVs including exosomes are the main targets of these studies (Figure 4), although misnaming is often evident due to lack of coherent isolation protocols, standardized terminology, and classification of EVs. For these reasons, authors will herein use the general term “EVs” when presenting study results. Indeed, the study of EVs is a new research field in VM, with a paramount potential in the “one health” perspective, but it is also a challenge for veterinary scientists as they need to gain more knowledge and expertise on EV isolation and analyses, with specific adaptation of protocols for animals, when needed. With this review, we hope that EV scientists will increase their awareness about the state of the art of EV research in the veterinary field, discovering potential common points and new possible applications of EVs among different species.

## 2. Critical Points of EV Research in Veterinary Medicine

Considering the recent appearance of EVs in VM research, some studies focused on the improvement of EV isolation and on their preliminary characterization from different animal samples.

Despite the first publication on EVs exploiting the use of animal cell culture [21], EV research in the following years proceeding exponentially only in human medicine, leading to the foundation of the International Society of EVs (ISEV) in 2011, to the first ISEV annual meeting in 2012 and to the first consensus guidelines (Minimal Information for Studies of Extracellular Vesicles) in 2014, updated in 2018 (MISEV 2018) [1,22]. Conversely, VM EV research stopped for many years and only in 2012 it started over with the first isolation of EVs from chicken dendritic cells (DCs). Consequently, in the following years, specific studies describing the successful isolation of EVs from canine, feline, and equine cell cultures were published [23,24,25].

More recently, technical issues have been addressed also referring to MISEV 2018, in which authors compared different purification methods, such as ultracentrifugation (UC), ultrafiltration and precipitation, size exclusion chromatography (SEC) and different starting samples, such as canine serum, feline plasma and urine, and equine uterine fluid [24,25,26,27,28].

However, standardization of protocols and terminology (i.e., MISEV 2018) are still major issues in EV research, and this is even more evident in VM.

The analysis of the reviewed papers reveals that the general main critical points of EV research in VM are the less rigorous selection of EV isolation and characterization techniques as described below.

**Figure 5:**
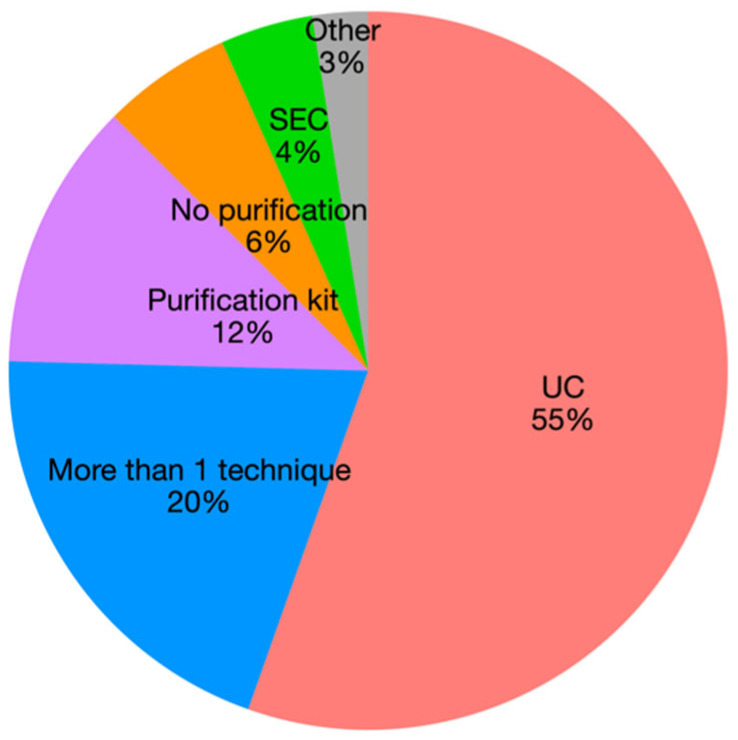
Pie chart representing the number of papers on EVs by purification method.

**Figure 6:**
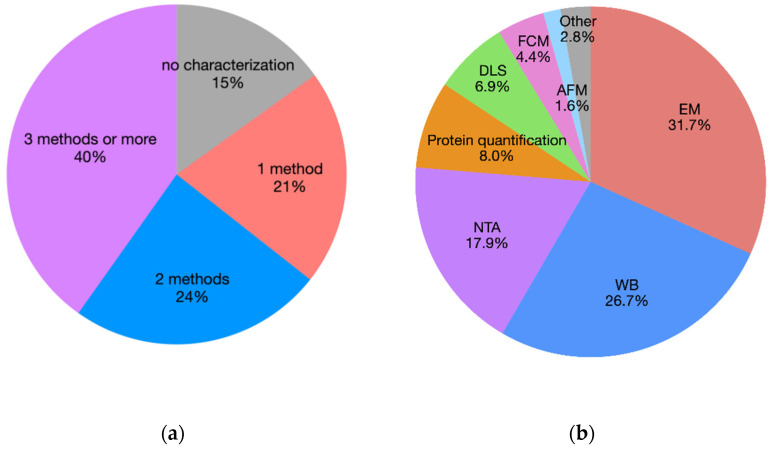
(**a**) Pie chart representing the percentage of papers on EVs by number of applied characterization techniques. (**b**) Pie chart representing the percentage of paper on EVs by characterization technique.

- Lack of standardization on EV isolation methods, often use of a single method: in the 80% of the reviewed papers a single technique is applied to isolate EVs, with only 20% of papers combining at least two methods. UC is the most commonly used, both as a single or combined technique, followed by commercial EV isolation kits (Figure 5). In addition, limitations of methods and controls are often not properly discussed or included.

- Lack of a constant, proper EV characterization: EVs are characterized in about 85% of the studies, and confirmation of the EV presence with at least two different methods is reported in 64% (Figure 6a). Several methods are used among which standard electron microscopy (EM) (32%), Western blot analysis (WB) (27%), and nanoparticle tracking analysis (18%) are the most commonly used (Figure 6b). However, in 15% of published papers (most of them published 2019–2021), no EV characterization is performed at all.

Several other critical aspects have emerged by the analysis of the VM literature related to the still limited number of studies performed on each animal species and the lack of standardization of the VM species-related EV knowledge, such as:

- Lack of a list of specific EV-related or -unrelated markers that can be used for EV characterization in different animal species;
- Lack of tissue- or cell-specific markers to isolate EV subpopulations;
- Lack of reference genes to be applied when EV-associated nucleic acids are investigated;
- Lack of species-specific pre-analytical indications. Often, veterinary scientists try to apply protocols and techniques derived from human (or sometimes lab animals) studies for the analysis of EVs in other animal species. However, there are several factors that can affect EV isolation and analysis, especially when body fluids are analyzed. The starting material (e.g., blood, saliva, urine, milk) can vary in composition depending on the animal species and this can imply differences in methods and protocols to analyze EVs.

**Figure 7:**
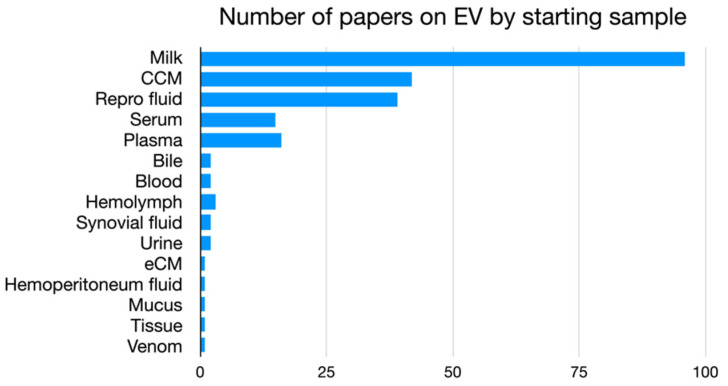
Bar graph representing the number of papers on EVs by starting sample.

The analysis of the VM literature shows that, after milk, reported as the most frequently addressed starting sample investigated for human applications, EVs are isolated from cell culture medium (CCM) in in vitro studies in 42 papers; other works analyzed EVs isolated from other body fluids, with the reproductive fluids (e.g., uterine flushing, follicular fluid, or semen) being the most represented (Figure 7).

**Figure 8:**
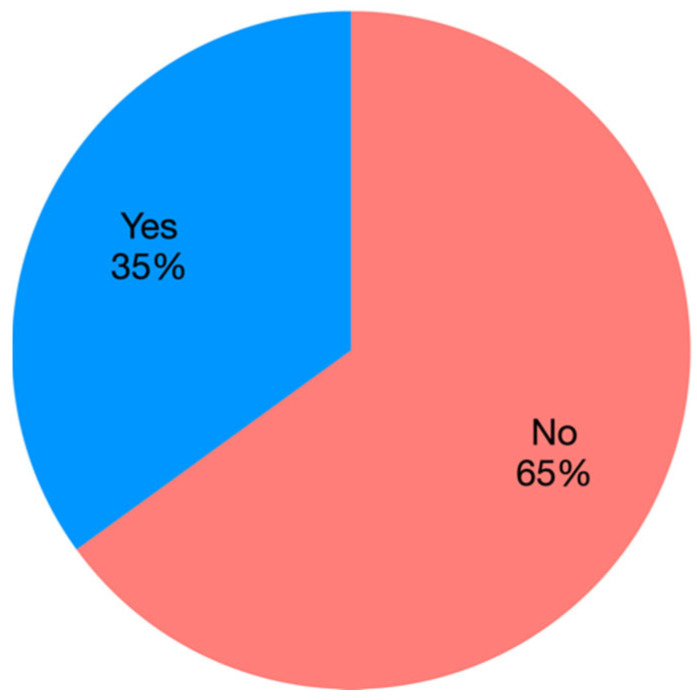
Pie chart representing the percentage of papers using EVs for functional studies.

Finally, research on EVs in VM is often descriptive, with a lack of functional studies. Functional studies are performed to test one or more specific function/s of the studied EVs and usually are based on in vitro or in vivo analyses in which EVs are uptaken by recipient cells. By the analyses of the selected papers, functional studies are limited to 35% and are mainly based on CCM-derived EVs for therapeutic applications (Figure 8).

Based on these considerations, this review presents in the following sections the major findings grouped per topic, selecting papers based on the following inclusion criteria:

- One internationally accepted (MISEV 2018) EV isolation/purification method;
- Minimum of two internationally accepted (MISEV 2018) methods for EV characterization.

Supplementary Table S1 includes the list of all papers that were revised for this work.

## 3. EVs in Physiology and Pathophysiology

EVs have an important role in intercellular communication both in physiological and pathological processes. Reviewing the VM literature on EVs, it was evident that major attention has been received by the function of EVs in animal immunity and reproduction. The former curiously includes the study of peculiar immune features which characterize animal species living in extreme environmental conditions, such as deep ocean or high altitudes, whereas studies on reproduction refer mainly to large animals, for obvious economic reasons. When it comes to EV roles in pathology, then infectious diseases take the stage since understanding their mechanisms of intra- and inter-species dissemination and of causing cell damage is of paramount importance for their control.

### 3.1. EVs in Immunity

Considering the rising impact of comparative immunology and the role of EVs in immunity, several animal-specific aspects of the immune system have been investigated with particular attention to the role of post-transcriptionally deiminated proteins contained within EVs [29].

Cartilaginous fishes have peculiar adaptive immune mechanisms and unusual lymphocyte antigen receptors which make them of interest for research in immunotherapeutic fields [30]. Criscitiello and colleagues isolated EVs from plasma of the nurse shark (*Ginglymostoma cirratum*) and assessed the presence of EV-derived deiminated proteins involved in immunity, i.e., hemopexin, a protein which contributes to heme homeostasis and also associated to physiological stress, haptoglobin, an acute phase protein and novel antigen receptor, a particular heavy chain homodimer with high target affinity and selectivity. Some of these EV proteins have been reported to be deiminated in cartilaginous fishes for the first time and further studies may highlight other interesting features of shark immunity [31].

A similar approach to the study of deiminated protein profiles of EVs has been conducted also on teleost fishes. As their mucosal immunity shares features with type I mucosal surfaces of mammals, Magnadottir and co-authors isolated EVs from pooled mucus collected from the dorsal side of farmed cods [32]. They assessed the presence of deiminated cytoskeletal proteins (i.e., elongation factor 1-alpha, fast skeletal muscle alpha-actin, profilin) and found in EV cargo also the complement component C3 and C-reactive proteins, suggesting the role of EVs in natural immunity against pathogens of the aquatic environment [32].

Other species of remarkable interest for comparative pathophysiology are crocodilians. Crocodilians have long life spans, low metabolic rates, strong antibacterial and antiviral abilities and are cancer resistant [33,34,35]. In alligators, EVs have been studied to analyze their role in the species strong immune resistance. EVs have been isolated from plasma of *Alligator mississippiensis* and their deiminated proteins cargo has been analyzed. Deiminated proteins were found to be involved in metabolism, gene regulation and cancer, providing new insights into the unique physiology of these species [35].

Marine mammals have undergone a range of physiological adaptations to diving and adapted their immune system to the aquatic environment. Research on their immune system may be useful to understand mechanisms such as resistance to cancer, insulin resistance, and adaptations to hypoxia, highly relevant for a number of human pathologies [36,37,38,39]. Marine mammals are of research interest also because their environment is changing, due to global warming, pollutant, and anthropogenic presence. These stress factors predispose them to resulting changes in exposure to pathogens and opportunistic infections with a possible virus-induced immunosuppression and increased bacterial and parasitic infections [40,41].

Therefore, as in humans, EVs could be studied to understand physiological/pathogenic pathways and to be exploited as diagnostic biomarkers to assess marine mammal health status.

In marine mammals, EVs have been isolated from serum of two seal species—gray seal (*Halichoerus gryptus*) and harbor seal (*Phoca vitulina*)—and of five cetaceans—northern minke whale (*Balaenoptera acutorostrata*), fin whale (*Balaenoptera physalus*), humpback whale (*Megaptera novaeangliae*), Cuvier’s beaked whale (*Ziphius cavirostris*), and orca (*Orcinus orca*) [42,43]. In these studies, the authors investigated specific EV-associated microRNAs and deiminated protein profiles. Three key miRNAs related to inflammation (miR-21), stress-response (miR-155), and hypoxia and metabolic activity (miR-210) were assessed and species-specific differences in the expression of these miRNAs were found. Deiminated proteins were detected in EVs by WB, indicating that EVs can mediate the transport of critical proteins for immunity and metabolism [42,43]. Additional findings also suggested that EVs could be a better source for the assessment of miRNA expression compared to whole serum, as in all the considered cetacean species the expression of the analyzed miRNA was considerably higher in EVs [43].

Not only marine mammals are exposed to anthropogenic pressures and climate change. Seabirds are the most threatened group of birds worldwide and finding biomarkers to assess their health status would be of paramount importance. EVs have been isolated from plasma of eight Antarctic seabird species: wandering albatross (*Diomedea exulans*), gray-headed albatross (*Thalassarche chrysostoma*), black-browed albatross (*Thalassarche melanophris*), northern giant petrel (*Macroneces halli*), southern giant petrel (*Macronectes giganteus*), white-chinned petrel (*Procellaria aequinoctialis*), brown skua (*Stercorarius antarcticus*), and south polar skua (*Stercorarius maccormicki*). In plasma derived-EVs of three of these species (the wandering albatross, the south polar skua, and the northern giant petrel), deiminated proteins belonging to immune and metabolic pathways were found and may become possible novel indicators of the immunological and physiological status of these animals [44].

### 3.2. EVs in Reproductive Physiology

Swine, bovine, and chicken are all species of agricultural importance and a better understanding of pregnancy mechanisms to improve outcomes is economically relevant. EVs have been studied mainly in swine and bovine to investigate their role in reproduction and to assess their function to improve reproductive outcome.

In swine, Bidarimath et al. demonstrated that EVs are released by porcine endometrium, chorioallantoic membrane, porcine trophectoderm cells (PTr2) and aortic endothelial cells (PAOEC) and that they contain proteins of signaling pathways relevant for angiogenesis and endometrial vasculature development, which are important processes in early pregnancy stages. In the same study, the potential ability of PTr2 and PAOEC EVs to regulate angiogenesis and the exchange of EVs between these two cell types have been demonstrated through proteomic analysis [45]. Female reproductivity has also been investigated through the analysis of EVs isolated from the follicular fluid (FF) of pigs [46,47]. Matsuno and co-authors evaluated mRNA profiles in EVs isolated from porcine FF compared to mRNAs extracted from mural granulosa cells (MGCs). They found that EVs in FF are mainly secreted by MGCs but also by other types of cells (e.g., tecal cells, oocytes, cells of non-ovarian tissues) and that the mRNAs content was involved in folliculogenesis pathways [47]. Grzesiak et al. identified and quantified FF EVs of small, medium, and large antral follicles of sexually mature gilts to conduct proteomic analysis of their cargo. More EVs were found in medium follicles compared to small and large follicles and proteomic analysis showed the presence of proteins mainly belonging to cytoskeleton or extracellular matrix, suggesting their role in building cell components and in follicular development [48].

In bovine, Gatien and collaborators analyzed the metabolomic profile of EVs isolated from the oviductal fluid (OF) and identified their metabolite content [49]. They found that the side of ovulation had no effect on EV metabolites concentration, but the reproductive cycle stage affected maltose, methionine, and glucose-1-phosphate levels in EVs. The pathways involving the analyzed metabolites were related to sucrose, glucose, and lactose metabolism [49]. Interestingly, bovine OF-derived EVs have also been demonstrated to maintain sperm survival and to stimulate processes associated with capacitation, therefore being possibly used to enhance assisted reproductive technologies [50]. OF-derived EVs can not only support sperm but also embryo cells. OF-derived EV supplementation has been demonstrated to increase survival rate of bovine blastocysts after vitrification and warming [51]. Moreover, embryo culture media-derived EVs can positively affect blastocysts, increasing the yield and reducing apoptosis [52]. RNA sequencing of bovine oviductal epithelial cells exposed in vitro to FF-derived Evs showed the presence of differentially expressed genes involved in sperm survival, fertilization, and embryo development [53]. Bovine FF Evs can also promote the synthesis of androstenedione and progesterone in ovarian cortical stromal cells and increase cell proliferation while inhibiting cell apoptosis on the same cells in vitro [54].

High temperatures, and consequent heat stress, are an issue for the reproductive management of dairy and beef cows. An in vitro model of bovine granulosa cells showed that Evs released by cells exposed to heat stress contained differentially expressed miRNAs. Moreover, the supplementation of heat stress-derived Evs to normal cells could induce an adaptive response to heat stress [55].

Few additional species are also included in some studies regarding Evs and reproduction.

In hens, the presence of Evs has been observed in the uterine fluid in vivo, and in sperm storage tubules in vitro [56,57]. Proteins involved in the regulation of sperm function were identified in uterine fluid-derived Evs, suggesting also the possible supportive role of Evs in avian sperm survival [56].

In boar, seminal plasma Evs have been analyzed and their RNA content was associated with immunity pathways which may establish the adequate uterine environment for fertilization and implantation [58].

In pets, a deeper understanding of reproductive mechanisms could be useful for the improvement of assisted reproductive technologies to preserve genetic traits [59]. Feline FF Evs contain proteins involved in follicle and oocyte maturation and can improve oocyte cryopreservation, enhancing their ability to resume meiosis [60]. The effects of feline and canine OF Evs on cryopreserved sperm has also been investigated and their ability to improve acrosome integrity of red wolf and cheetah sperm and red wolf sperm motility has been demonstrated in vitro [61].

### 3.3. Evs in Pathophysiology of Infectious Diseases

Infectious diseases and zoonoses are some of the main issues in the management of farm animals. Understanding and controlling infective agents is of pivotal importance for animal health and welfare, food safety, and to keep a high productivity, especially in developing countries [15]. In human medicine, many studies showed that human EVs can carry viral elements, being both involved in the spread of infectious diseases and in the induction of an immune response against the infective agent [62,63,64].

EV-related miRNAs have been proven to be differentially expressed in infected humans, therefore becoming tools to better understand the pathogenic mechanism of a disease or useful diagnostic biomarkers [65].

In VM, some evidence has been demonstrated of the role of EVs as regulators of viral transmission. An example is a study performed on the seminal plasma of cocks infected with subgroup J of Avian Leukosis Virus (ALV-J). Nine EV-related miRNAs were found to be differentially expressed: 3 upregulated and 6 downregulated. Among these miRNAs, the tumor suppressor miR-138-5p was found to be downregulated as in many types of human cancer [66].

Other important viral diseases in farm animals have been preliminarily approached. EVs released by HeLa cells infected with Newcastle Disease Virus (NDV), or by Mardin-Darby bovine kidney cells (MDBK) infected by Caprine parainfluenza virus type 3 (CPIV3), were demonstrated to carry miRNAs, RNAs, or proteins which enhanced the cytopathic effect of the virus. Furthermore, EVs were also able to suppress interferon (IFN)-beta gene expression in HeLa cells after NDV infection, and to inhibit autophagy during CPIV3 infections, suggesting a significant role of EVs in viral spreading [67,68].

EVs are also involved in long term persistence of some viruses [69,70]. miRNA eca-mir-128 which targets CXCL16, the gene involved in the persistence of Equine Arteritis Virus (EAV) infection, was detected in seminal plasma EVs of infected stallions [71].

Understanding the pathogenesis of infectious diseases is an essential step also for their management and prevention. Bovine leukemia virus (BLV) often causes asymptomatic infection and infected animals may remain asymptomatic virus carriers for their entire life [72]. Even if considered a minor route of infection, lactation may transmit BLV particles and BLV-infected somatic cells to calves [73]. Yamada et al. showed that EVs isolated from milk of infected cattle contain BLV structural proteins, but these proteins did not seem to be infectious to cells after in vitro analysis, as proviral DNA was not detected [74]. Montaner-Tarbes and collaborators found the presence of viral proteins in EVs isolated from the serum of pigs infected with African Swine Fever virus. These proteins could be exploited for EV-based vaccines, similarly to what the same authors performed in a trial against Porcine Reproductive and Respiratory Syndrome virus (PRRSV) (see below) [75,76].

### 3.4. EVs in Environmental Adaptation and Nutrition

Other processes in which animal EVs have been investigated mainly deal with metabolism. In this regard, some animal species have peculiar adaptive mechanisms for living in low oxygen milieu [77]. Among these species, the naked mole-rats are among the most hypoxia-tolerant mammals and this phenomenon is related to their ability to reduce energy (O_2_) supply, decreasing their metabolic rate up to 85% [78,79]. Naked mole-rats also have several other adaptive abilities, such as a remarkable longevity and resistance to cancer; they are also the only thermo-conformers mammalian almost completely ectothermic for body temperature regulation [80,81,82]. These adaptive and immune features make the naked mole-rat an interesting animal model for human diseases and for understanding cancer resistance and longevity pathways. In 2019, Pamenter and collaborators profiled plasma-derived EVs in naked mole-rats to assess the presence of deiminated proteins and stress/hypoxia related miRNAs (e.g., miR-21, miR-155, and miR-210). Interestingly, several deiminated proteins related to glycolysis, Hyopxia-Inducible Factor-1 (HIF-1) pathway, and also targeted miRNAs were all expressed in EVs, indicating their possible role in such an extreme adaptive capability [83].

Camelids are also species of relevant interest for their adaptive strategies [84]. Genomic analysis showed that they have unique adaptive features related to fat and water metabolism, heat stress response, and oxygen transport allowing them to live in extreme environments such as the desert and the high altitudes [84,85]. Because of their adaptive strategies, camelid physiology has been considered useful for human research so that more in depth examinations have recently started involving EVs. In 2020, Criscitiello and colleagues analyzed deiminated proteins in llama (Lama glama) serum and in serum-derived EVs. They found post translational modification in proteins involved in metabolism and immunity and a partially overlapping of deiminated proteins profile between serum and serum-derived EVs, with some proteins identified only in serum and others only in EVs. In llamas, some of these proteins were reported as post-translationally deiminated for the first time, and some of them are known to be involved in human diseases (e.g., dystonin, Xaa-Pro dipeptidase). Further comparative studies may deepen our understanding on the regulation process of these proteins and, consequently, of correlated human diseases [86]. Finally, similarly to sharks, camelids produce small homodimeric heavy chain-only antibodies with variable binding domains that can be highly useful in medicine and biotechnology research applications because of their small size, economic production, specificity, affinity, and stability [30,87,88].

Another curious finding in exotic species is the evidence of EVs in snake venom. Studies on snake venom EVs were conducted to understand their role in the envenomation process and as preliminary analysis to find new future targets for the development of anti-venoms compounds. Snake venom EVs carry proteins which are normally present in snake venom and are functionally active, with fibrinogenolytic and cytotoxic activity [89].

Further, a nutritional role of EVs has been preliminarily investigated in animals. One of the main implications of studying dietary EVs is related to their bioavailability and to the possibility of absorbing their content [90]. A recent study assessed the potential postprandial transfer of bovine colostral EVs and of EV-associated cargo to newborn calves, evaluating the presence of specific colostral proteins and miRNAs in calf circulation. While colostral EV protein markers were detected in calves’ post-prandial blood, only a little overlapping of miRNA expression profile was present. These findings may suggest a different uptake according to the localization of EV components, since the investigated proteins were EV membrane-bound whilst miRNAs are usually mainly in the EV lumen [91].

Among avian species, pigeons have the unique ability to produce a nutrient substance which resembles bovine milk. EVs have been therefore isolated from pigeon ‘milk‘ and EV-related miRNA analyses showed that 10 miRNAs are co-expressed in 5 different stages of ‘lactation‘. They were mainly related to immunity and growth. Moreover, 81 miRNAs were commonly shared with milk of other mammalian species [92].

Finally, an interesting study performed on bovine serum EVs showed that EV cargo can also be influenced by nutrition. The administration of different diets was associated with the presence of differentially expressed miRNAs related to hormone pathways and protein metabolism [93].

## 4. EVs as Biomarkers

One of the critical aspects of using EVs as biomarkers, as for any other biomarker, is the need for standardization [94,95].

An interesting preliminary study of Narita and collaborators compared three different EV isolation techniques (UC, membrane affinity chromatography, and precipitation) on canine plasma to find reference genes for EV-related miRNAs [96]. The authors found that UC was the most stable method and identified miR-103 as the most reliable miRNA to normalize miRNAs expression [96]. However, many studies suffer technical biases and lack of standardization and details should be carefully assessed. The main findings in VM literature follow.

### 4.1. EVs as Biomarkers in Reproduction

Hua and collaborators found small RNAs in swine uterine fluid-derived Evs of gilts on days 10, 13, and 18 of pregnancy that were involved in pathways of immunization, endometrial receptivity, embryo development, and implantation. They also highlighted a different expression of some miRNAs during the three phases of pregnancy, whose role as biomarkers to monitor pregnancy could be further studied [97].

Other studies focused on the study of EVs to improve the efficiency of early pregnancy diagnosis in swine. Two miRNAs, miR-92b-3p and miR-15-5p, were found to be upregulated in serum-derived EVs of sows in days of pregnancy 9, 12, and 15, being possible circulating biomarkers to diagnose early pregnancy [98].

In plasma-derived EVs of goats, 5 miRNAs were found exclusively in pregnant animals, being candidates for early pregnancy diagnosis in this species [99].

Embryo-derived EVs could also be useful to indicate cell status and to help select competent blastocysts. In bovine, Mellisho and collaborators demonstrated that bovine blastocysts secrete EVs in the culture media and that the EVs concentration and diameter are different whether embryos are produced by in vitro fertilization or by parthenogenetic activation [100]. Melo-Braez and colleagues also found that miRNA content in EVs released by bovine embryos during their compaction period was different whether embryos were competent and reached blastocyst stage or arrested in the 8–16 cells stage [101].

Finally, the possibility to differentiate EVs according to their origin has been assessed on bovine serum by Raman spectroscopy allowing distinction of placental EVs from mononuclear cell EVs [102]. Raman spectroscopy takes advantage of laser light scattering due to photons interactions to provide information on the biochemical components of a sample [103]. In bovine serum, different clusters, implying a difference in EV cargo composition and origin, were identified. Further, differences in cargo composition were present also in EVs from different pregnancy stages, suggesting that Raman spectroscopy could be a useful tool to monitor placental development [102].

### 4.2. EVs as Biomarkers in Oncology

Tumor cells produce a high amount of EVs, who take part in every step of tumor progression and whose cargo is related with the stage and the grade of the tumor [104]. These factors highlight the importance of EVs as biomarkers for cancer diagnosis, prognosis, and therapy.

Cancer is one of the main causes of death also in companion animals, which are considered spontaneous models in comparative oncology [105]. Biomarkers are sought for many cancer types also in pets and recently, EVs have become an interesting research target in this field, even if few studies are available. However, standardization of procedures is of remarkable importance in this contest and should be dramatically optimized in VM [106].

MiRNAs are important cancer biomarkers carried by EVs and are largely studied in human medicine for their possible use in liquid biopsy in particular for early tumor diagnosis [107,108,109]. Studies performed in vitro on canine cell lines provided the first evidence of different EV-related miRNA contents in normal canine mammary epithelial cells versus tumor canine mammary epithelial cells [110]. The benefit of miRNA analysis in tumor diagnosis was also investigated in vivo by Narita and collaborators who found that miR-15b and miR-342-3p derived from plasma EVs could be a potential non-invasive biomarker to differentiate dogs with glioblastoma from dogs with other brain diseases [111].

EV-miRNAs expression can also be exploited for prognostic aims [112,113]. In one clinical trial performed on dogs with multicentric lymphoma, four miRNAs in plasma-derived EVs were found to be differentially expressed between dogs with progressive disease and dogs that reached complete remission [114].

Together with diagnosis and prognosis, the outcome of a chemotherapeutic protocol is another important issue in oncology [115,116]. In vitro studies performed on EVs from canine lymphoma cell lines evidenced the presence of three miRNAs which were differentially expressed between vincristine sensitive and vincristine resistant cell lines [117]. In another study in dogs with long term treatments with doxorubicin, plasma EV-related miRNAs were demonstrated to be efficient in the diagnosis of cardiotoxicity. Indeed, 3 miRNAs were differentially expressed in dogs with cardiotoxicity and miR-502 was upregulated before the third chemotherapeutic dose, being an earlier biomarker than cardiac troponin I (cTnI) [118].

### 4.3. EVs as Biomarkers for Other Diseases

The use of EV-related miRNAs as biomarkers has been explored also for the diagnosis of other diseases than cancer.

In canine urinary EVs, a pool of 5 miRNAs has been compared in dogs with healthy kidney versus dogs with kidney diseases. miR-26a, miR-10a/b and miR-191 were differentially expressed, suggesting that further studies could assess the role of urine EVs in the diagnosis of kidney dysfunction, as largely demonstrated in humans [119,120,121].

The first publications on EVs in animals were related to the fate and metabolism of the transferrin receptor (TfR) after endocytosis in cells for iron uptake [10,11,122]. More recently, other researchers focused again on the presence of TfR on EVs membrane, evaluating its role as biomarker for anemia in horses, dogs, and cats [123,124]. In six horses with regenerative anemia, TfR1 expression on serum-derived EVs increased together with the progression of the disease [123]. Another study performed on horses, dogs, and cats focused instead on the proportion of cytoplasmic domain of TfR (cTfR) and total TfR on serum-derived EV membrane to calculate indirectly the presence of soluble TfR (sTfR), the latter used as biomarker for iron deficiency in humans. No difference between healthy and diseased animals was found in the expression of cTfR, suggesting that quantification of sTfR in animals might be less useful to diagnose iron deficiency during inflammatory disease than in humans [124].

Musculoskeletal and joint diseases are the most common health issue and cause of wastage in the equine industry [125]. However, joint diseases in horses are not only of interest for the veterinary field but also for human medicine, as the horse is considered a good model for human orthopedic research because of the similarities of joints and cartilage [126,127,128]. Therefore, research on synovial fluid (SF)-derived EVs to unravel both their physio-pathological role and their potential as biomarkers in joint diseases has recently started [129]. Although EV isolation from SF has not been much explored, preliminary research to optimize EV isolation from SF has been performed on horses. SF is a fluid characterized by high viscosity and it has been demonstrated that hyaluronidase pretreatment of SF prior to UC facilitated the recovery of CD44+ EVs [130].

## 5. EVs in Therapy

Studies regarding the use of EVs in animal therapy are not many and still preliminary.

In pets, only one paper focused on the use of EVs in cancer therapy in vitro. EVs derived from canine M1-polarized macrophages were administered in vitro to canine osteosarcoma and melanoma cell lines. EVs treatment increased the level of pro-inflammatory cytokines and induced apoptosis of tumor cells [131].

Stem cells have attractive biological features which are of interest both for human and veterinary research [132]. Since stem cells have regenerative effects, stem cell-derived EVs are largely studied for therapeutic applications [133].

In a veterinary clinical trial, a horse with a suspensory ligament injury was treated with EVs isolated from adipose-derived stem cells combined with 5-azacytydine and reseveratrol. EV treatment was promising, causing lesion filling, angiogenesis, and tissue elasticity [134].

The effect of mesenchymal stem cells (MSC)-derived EVs has been tested in vitro in some studies performed on equine and canine cells. EVs isolated from equine adipose MSCs and from canine MSCs demonstrated to have anti-inflammatory effects in vitro on recipient endometrial and glial cells, respectively [135,136].

In an in vivo mouse model, canine adipose tissue derived MSC-derived EVs were administered intraperitoneally to mice with dextran sulfate sodium-induced colitis. In mice treated with EVs, inflammation was alleviated, although the injection of TSG-6-depleted EVs reduced this effect. Particularly, the authors confirmed through immunofluorescence on colon samples that TSG-6 in EVs enhanced regulatory T cells and the polarization of macrophages from M1 to M2 [137].

Additionally, a commercial preparation of human plasma-derived EVs was able to increase cell proliferation and collagen deposition in vitro on a canine tenocyte culture. Moreover, EV treatment reduced cellular apoptosis caused by dexamethasone on the same canine cell line [138].

## 6. EVs as Vaccines

Due to the rise in worldwide population expected in the next decades, a strong increase in global food production is needed, including food of animal origin. In parallel, an enhanced attention for animal welfare issues is likely to increase the expenditure on pet supplies as well as the search for innovative medical approaches. Further, the role of animals in the “one health” perspective and in spreading of novel infections has become more evident. For all these reasons, development of veterinary vaccines is receiving stronger attention and economic investments. At the same time, novel approaches for vaccine development are required, since classical and live-attenuated vaccines are not totally safe and efficacious [139].

Up to November 2021, three papers on avian species and two papers on swine have been published on the use of EVs as vaccines.

Considering pork industries, one of the main threats is the Porcine Reproductive and Respiratory Syndrome Virus (PRRSV) caused by an enveloped RNA virus of the Arterivirus genus, first described in the US in 1987–1988 and referred to as mystery pig disease [140]. Since then, it has become one of the most important swine diseases of the last half-century causing reproductive impairment and pneumonia with no single successful strategy for control [141,142]. As for other diseases, vaccine development has therefore received wide attention. Current available vaccines against PRRSV have a series of limitations such as partial protective immunity, possible reversion to virulence with consequent biosafety problems, inability to induce long lasting and heterologous protection, and high antigenic and genetic differences of strains [76,143]. Interestingly, in 2016, Montaner-Tarbes and collaborators demonstrated that in the serum of animals that had overcome a PRRSV infection there were EVs free of virus but containing viral proteins with immunogenic properties [143]. In 2018, they reported the first trial to immunize pigs with Evs isolated from serum of pigs which had suffered a natural PRRSV infection. They demonstrated that EV-enriched fractions obtained by SEC were free of virus, safe, and with viral peptides capable of eliciting humoral and cellular immune responses with no adverse immune reaction. Moreover, in pigs vaccinated with Evs followed by boosts with synthetic peptides, the authors recorded a specific humoral IgG response which may be useful to differentiate vaccinated from infected animals [76].

The use of EVs as a vaccine was tested also In poultry against coccidiosis. Coccidiosis is a group of parasitic diseases caused in poultry by protozoans of the gender *Eimeria* spp. The infection causes mainly weight loss and poor feed conversion ratio with consequent losses in poultry production [144]. A preliminary trial evaluated the immunization caused by eVs isolated from chicken intestinal dendritic cells (DC) stimulated in vitro with a mixture of *Eimeria* antigens. In vaccinated animals, a T-cell immune response against *Eimeria* was demonstrated [145]. However, isolation of eVs from DCs (or any other cell type) on a large scale for massive vaccinations was considered difficult to practice. Considering serum as a more convenient source of eVs, the protective ability of serum-derived eVs obtained from *E. tenella* infected chickens was tested. A protective immunity was recorded in vaccinated animals, with reduced gut lesions and parasite shedding, increased weight gain, and improved feed efficiency [146]. Moreover, considering the importance of CD80 in antigen presentation, the authors demonstrated that immunity improved when selected CD80+ eVs were administered compared to vaccination with CD80- eVs [146].

The application of eVs for antigen delivery is now very promising in human medicine both for anti-cancer immunotherapy and for conventional prophylactic application against infectious diseases including SARS-CoV-2 [147]. More advances should be made also in VM considering the tremendous role of large-scale vaccination in animals for both animal and public welfare.

Preliminary studies in VM also explored the possible use of eVs, and particularly their miRNAs cargo, as biomarkers to distinguish vaccinated by non-vaccinated animals. Vaccination for Marek’s Disease (MD) in chickens, a viral disease causing the development of multiple lymphoid tumors, was associated with an increased expression of tumor suppressor miRNAs in serum eVs. Moreover, in tumor-bearing chickens more EV-related onco-miRNAs were reported, with gg-miR-146a and -21 being good candidates to distinguish non-vaccinated tumor-bearing animals from vaccinated animals [148]. Further studies demonstrated that in serum eVs of chickens vaccinated for MD, mRNA mapping the whole genome of MD virus was present, suggesting the participation of eVs in vaccine immune response [149].

## 7. EVs from Animal Milk for Human Applications

EVs in human breast milk were first identified in 2007 by Admyre and collaborators who described their immunological potential [150]. In the last 15 years, nearly 100 publications explored eVs in milk from several animal species, mainly focusing on bovine milk, considered an abundant, cost-effective, and biocompatible source of eVs for human medicine research [151]. A non-exhaustive summary is here proposed with regard to the main applications and findings.

Milk is a complex fluid for EV isolation, particularly rich in proteins that may interfere with purification steps. Among these proteins, casein micelles have size and density comparable to eVs, and thus can be co-isolated with milk-derived eVs (MEVs). For this reason, many studies focused on the implementation of MEV purification protocols, which however are not yet properly standardized [152,153]. Preliminary centrifugation steps are used to remove fat globules and casein aggregates and, indeed, centrifugation combined to SEC have been successfully applied for bovine MEVs (bMEVs) purification [154,155,156]. Recently, it has been demonstrated that adding hydrochloric acid or acetic acid to milk can accelerate casein aggregation and precipitation, facilitating MEV purification and separation from casein, even if a partial degradation of EV-surface proteins has been observed [157,158,159].

Since it has been demonstrated that MEVs (and their RNA content) can resist digestion, one of the biological relevance of milk lies in the possibility that recipient host cells can uptake viable MEVs and their cargo [160,161]. Although a recent study has demonstrated that many miRNAs in human, bovine, and caprine milk are preserved across species, interspecies MEVs dietary uptake could still result in an exchange of RNA which may regulate host gene expression, despite their low concentration [162,163,164].

In the last few years scientists have started to investigate the interspecies effects of orally administered bMEVs, primarily on intestinal health and integrity. In vitro studies performed on intestinal crypt epithelial cells exposed to oxidative stress, showed the protective effect of bMEVs pretreatment, with a consequent reduction of reactive oxygen species [165]. In vivo oral administration of bMEVs to mice has demonstrated to have effects on gut microbiota composition and to improve local intestinal immunity, increasing the expression of genes important for mucosal integrity [166]. The positive effect of bMEVs on intestinal integrity has been assessed also in other studies, where they have been tested in models of malnutrition, ulcerative colitis, and necrotizing enterocolitis. In all these studies, bMEV were able to cause a protective or an alleviated effect on treated mice [167,168,169,170,171].

Milk and dairy products contain components associated with bone formation and maintenance, in particular if assumed during childhood and adolescence [172]. Recently, few studies also investigated the role of bMEVs on bone health [173,174,175]. In vitro studies on human osteoblastic Saos-2 and pre-osteoblastic MC3T3-E1 cells, demonstrated that bMEVs promoted cell proliferation and differentiation [173]. Moreover, in the same study, bMEVs oral administration to rats, enhanced osteogenesis, increasing tibial longitudinal growth and mineral density [173]. Other in vivo studies performed in mice models also showed that oral administration of bMEVs was able to improve bone mineral density in osteoporosis and was osteoprotective and able to reduce osteoclast presence in mice ovariectomized or with diet-induced obesity [174,175].

As well as the positive effects of bMEVs intake, some scientists also investigated the possible negative consequences of dietary assumption. In one study, it was observed that bMEV-associated miR-148 k seems to have a diabetogenic effect, promoting pancreatic β-cell differentiation to a more immature metabolic phenotype. This can affect insulin secretion and cause β-cell apoptosis [176].

The interest in bMEVs is also related to their anticancer properties. At proteomic analysis, Fonseka and collaborators showed that the incubation of bMEVs with neuroblastoma (NBL) cells significantly attenuated proliferation, confirmed by depletion of proteins implicated in proliferation, cell cycle, and Wnt signaling pathway and enrichment in proteins implicated in apoptosis and cellular senescence [177]. Moreover, in the same study, bMEV administration combined with doxorubicin increased the sensitivity of NBL cells to doxorubicin [177].

In oncology, bMEVs could also be exploited as drug carriers. MEVs are scalable, safe, cost-effective nanocarriers able to deliver a wide range of drugs, small molecules as well as macromolecules [151,178]. Drug-loaded bMEVs can also be engineered on their surface to target specific tumor cells, to improve therapeutic efficacy and reduce toxicity [151]. In an in vitro study, bMEVs were engineered to express hyaluronan (HA) on their surface and loaded with doxorubicin. HA is a specific ligand for CD44, a stem-like cell receptor usually overexpressed on the cell surface of many cancer subtypes. Results demonstrated that HA-bMEVs triggered tumor cell death showing the efficacy for tumor specific drug delivery [179]. bMEVs surface can also be engineered to facilitate their transit. bMEVs coated with polyethylene glycol had enhanced resistance to stomach acid environment and improved permeability to mucin, while delivering functional siRNA [180].

bMEVs may be useful also for dairy herd health management. Cai and collaborators characterized the different expression pattern of miRNAs in bovine bMEV from normal healthy cattle and from milk of cattle affected by mastitis. They found 18 miRNAs involved in immunity, with different expressions between the two groups [181].

Even if most of the studies on MEVs have been performed on bovine milk, there are some exceptions, with studies focusing on milk of other species. Metabolomic and transcriptomic analysis performed on the cargo of MEVs of donkeys and goats showed the presence of metabolites (e.g., arginine, asparagine, glutathione and lysine) and miRNAs with immunomodulatory properties [182,183]. Preliminary studies have been performed on yak milk and in vitro assays demonstrated that yak MEVs can alleviate intestinal inflammation and hypoxia consequences, promoting intestinal cell survival in a rat model [184,185]. Some studies have also been performed on milk not for human consumption, such as porcine milk [186]. Similarly, also the effect of porcine MEVs on the gastro-enteric tract has been evaluated. In particular, MEVs have been demonstrated to be protective against damages caused by the mycotoxin deoxynivalenol. This protective effect has been shown both in in vitro and in vivo studies, where porcine MEVs were able to promote intestinal cell proliferation and to inhibit cell apoptosis in mice [186].

## 8. Conclusions

The EV research in VM is at an early stage. However, more than 220 papers have been published counting until November 2021, mainly in the last few years, investigating many different areas of EV research in physiology and pathophysiology, as well as potential EV applications as biomarkers or therapeutic tools. All these papers give a glimpse of the enormous potential scientific impact of studying EVs in VM and on the important consequent implications. Furthermore, veterinary researchers are enthusiastic in studying EVs, their diverse roles, and potential applicability in animals, with the main aim of contributing to a better understanding of EVs biology and functions, but sometimes with too little background on EV topic and related technical issues. We strongly believe that a tight collaboration should be encouraged between emerging EV veterinary researchers and specialized EV scientists, in order to decrease technical biases and weaknesses and to get the most from common EV research.

## Data Availability

All data supporting reported results can be found in the Supplementary Table.

## Supplementary Materials

The following supporting information can be downloaded at: https://www.mdpi.com/article/10.3390/ani12192716/s1, Table S1: list and features of all the references on extracellular vesicles in veterinary medicine.
