# Supplementary material for: Extracellular Vesicles in Veterinary Medicine
Source: Animals (Basel). 2022 Oct 10;12(19):2716. doi: 10.3390/ani12192716 (PMC9559303; doi:10.3390/ani12192716)
Supplement: Supplementary file 1 [file animals-12-02716-s001.zip › Table_references.pdf]

1. Alvarez-Rodriguez, M.; Ntzouni, M.; Wright, D.; Khan, K.I.; López-Béjar, M.; Martinez, C.A.; Rodriguez-Martinez, H. Chicken Seminal Fluid Lacks CD9- and CD44-Bearing Extracellular Vesicles. *Reprod. Domest. Anim.* **2020**, *55*, 293–300, doi:10.1111/rda.13617.

2. del Cacho, E.; Gallego, M.; Lillehoj, H.S.; Quilez, J.; Lillehoj, E.P.; Sánchez-Acedo, C. Tetraspanin-3 Regulates Protective Immunity against Eimeria Tenella Infection Following Immunization with Dendritic Cell-Derived Exosomes. *Vaccine* **2013**, *31*, 4668–4674, doi:10.1016/j.vaccine.2013.06.109.

3. Hong, Y.; Lee, J.; Vu, T.H.; Lee, S.; Lillehoj, H.S.; Hong, Y.H. Immunomodulatory Effects of Poly(I:C)-Stimulated Exosomes Derived from Chicken Macrophages. *Poult. Sci.* **2021**, *100*, 101247, doi:10.1016/j.psj.2021.101247.

4. Huang, A.; Isobe, N.; Yoshimura, Y. Changes in Localization and Density of CD63-Positive Exosome-like Substances in the Hen Oviduct with Artificial Insemination and Their Effect on Sperm Viability. *Theriogenology* **2017**, *101*, 135–143, doi:10.1016/j.theriogenology.2017.06.028.

5. Liu, E.; Sun, X.; Wang, X.; Wang, T.; Li, W.; Tarique, I.; Yang, P.; Chen, Q. In Vivo Dynamic Distribution of Multivesicular Bodies and Exosomes in Spleen of DTMUV Infected Duck. *Vet. Microbiol.* **2019**, *229*, 138–146, doi:10.1016/j.vetmic.2018.12.014.

6. Cordeiro, L.; Riou, C.; Uzbekov, R.; Gérard, N. Avian Sperm Increase in Vitro the Release of Exosomes from SST-Enriched Organoids. *Reproduction* **2021**, *161*, 375–384, doi:10.1530/REP-20-0421.

7. O'dowd, K.; Emam, M.; El Khili, M.R.; Emad, A.; Ibeagha-Awemu, E.M.; Gagnon, C.A.; Barjesteh, N. Distinct MiRNA Profile of Cellular and Extracellular Vesicles Released from Chicken Tracheal Cells Following Avian Influenza Virus Infection. *Vaccines* **2020**, *8*, 438, doi:10.3390/vaccines8030438.

8. Wang, Y.; Wang, G.; Wang, Z.; Zhang, H.; Zhang, L.; Cheng, Z. Chicken Biliary Exosomes Enhance CD4+T Proliferation and Inhibit ALV-J Replication in Liver. *Biochem. Cell Biol.* **2014**, *92*, 145–151, doi:10.1139/bcb-2013-0096.

9. Ye, F.; Wang, Y.; He, Q.; Cui, C.; Yu, H.; Lu, Y.; Zhu, S.; Xu, H.; Zhao, X.; Yin, H.; et al. Exosomes Transmit Viral Genetic Information and Immune Signals May Cause Immunosuppression and Immune Tolerance in ALV-J Infected Hd11 Cells. *Int. J. Biol. Sci.* **2020**, *16*, 904–920, doi:10.7150/ijbs.35839.

10. Zhou, D.; Xue, J.; He, S.; Du, X.; Zhou, J.; Li, C.; Huang, L.; Nair, V.; Yao, Y.; Cheng, Z. Reticuloendotheliosis Virus and Avian Leukosis Virus Subgroup J Synergistically Increase the Accumulation of Exosomal MiRNAs. *Retrovirology* **2018**, *15*, 45, doi:10.1186/s12977-018-0427-0.

11. Capomaccio, S.; Cappelli, K.; Bazzucchi, C.; Coletti, M.; Gialletti, R.; Moriconi, F.; Passamonti, F.; Pepe, M.; Petrini, S.; Mecocci, S.; et al. Equine Adipose-Derived Mesenchymal Stromal Cells Release Extracellular Vesicles Enclosing Different Subsets of Small RNAs. *Stem Cells Int.* **2019**, *2019*, 4957806, doi:10.1155/2019/4957806.

12. Da Silveira, J.C.; Carnevale, E.M.; Winger, Q.A.; Bouma, G.J. Regulation of ACVR1 and ID2 by Cell-Secreted Exosomes during Follicle Maturation in the Mare. *Reprod. Biol. Endocrinol.* **2014**, *12*, 44, doi:10.1186/1477-7827-12-44.

13. Lange-Consiglio, A.; Lazzari, B.; Perrini, C.; Pizzi, F.; Stella, A.; Cremonesi, F.; Capra, E. MicroRNAs of Equine Amniotic Mesenchymal Cell-Derived Microvesicles and Their Involvement in Anti-Inflammatory Processes. *Cell Transplant.* **2018**, *27*, 45–54, doi:10.1177/0963689717724796.

14. Marędziak, M.; Marycz, K.; Lewandowski, D.; Siudzińska, A.; Śmieszek, A. Static Magnetic Field Enhances Synthesis and Secretion of Membrane-Derived Microvesicles (MVs) Rich in VEGF and BMP-2 in Equine Adipose-Derived Stromal Cells (EqASCs)—a New Approach in Veterinary Regenerative Medicine. *Vitr. Cell. Dev. Biol. - Anim.* **2015**, *51*, 230–240, doi:10.1007/s11626-014-9828-0.

15. Marycz, K.; Michalak, I.; Kocherova, I.; Edziak, M.M.; Weiss, C. The Cladophora Glomerata Enriched by Biosorption Process in Cr(III) Improves Viability, and Reduces Oxidative Stress and Apoptosis in Equine Metabolic Syndrome Derived Adipose Mesenchymal Stromal Stem Cells (ASCs) and Their Extracellular Vesicles (MV's). *Mar. Drugs* **2017**, *15*, 385, doi:10.3390/md15120385.

16. Andriessen, A.; Bongiovanni, L.; Driedonks, T.A.P.; van Liere, E.; Seijger, A.; Hegeman, C. V.; van Nimwegen, S.A.; Galac, S.; Westendorp, B.; Nolte-'t Hoen, E.N.M.; et al. CDC6: A Novel Canine Tumour Biomarker Detected in Circulating Extracellular Vesicles. *Vet. Comp. Oncol.* **2021**, 381–392, doi:10.1111/vco.12781.

17. Avenick, D.; Kidd, L.; Istvan, S.; Dong, F.; Richter, K.; Edwards, N.; Hisada, Y.; Posma, J.J.N.; Massih, C.A.; Mackman,

N. Effects of Storage and Leukocyte Reduction on the Concentration and Procoagulant Activity of Extracellular Vesicles in Canine Packed Red Cells. *J. Vet. Emerg. Crit. Care* **2021**, *31*, 221–230, doi:10.1111/vec.13050.

18. Lee, S.H.; Lira-Albarrán, S.; Saadeldin, I.M. Comprehensive Proteomics Analysis of in Vitro Canine Oviductal Cell-Derived Extracellular Vesicles. *Animals* **2021**, *11*, 573, doi:10.3390/ani11020573.

19. Park, S.-M.; An, J.-H.; Lee, J.-H.; Kim, K.-B.; Chae, H.-K.; Oh, Y.-I.; Song, W.-J.; Youn, H.-Y. Extracellular Vesicles Derived from DFO-Preconditioned Canine AT-MSCs Reprogram Macrophages into M2 Phase. *PLoS One* **2021**, *16*, e0254657, doi:10.1371/journal.pone.0254657.

20. Rowlison, T.; Cleland, T.P.; Ottinger, M.A.; Comizzoli, P. Novel Proteomic Profiling of Epididymal Extracellular Vesicles in the Domestic Cat Reveals Proteins Related to Sequential Sperm Maturation with Differences Observed between Normospermic and Teratospermic Individuals. *Mol. Cell. Proteomics* **2020**, *19*, 2090–2103, doi:10.1074/mcp.RA120.002251.

21. Rowlison, T.; Ottinger, M.A.; Comizzoli, P. Exposure to Epididymal Extracellular Vesicles Enhances Immature Sperm Function and Sustains Vitality of Cryopreserved Spermatozoa in the Domestic Cat Model. *J. Assist. Reprod. Genet.* **2021**, 25–27, doi:10.1007/s10815-021-02214-0.

22. Šimundić, M.; Švara, T.; Štukelj, R.; Krek, J.L.; Gombač, M.; Kralj-Iglič, V.; Tozon, N. Concentration of Extracellular Vesicles Isolated from Blood Relative to the Clinical Pathological Status of Dogs with Mast Cell Tumours. *Vet. Comp. Oncol.* **2019**, *17*, 456–464, doi:10.1111/vco.12489.

23. Sowy, S.; Rutter, C.R.; Jeffery, U. Extracellular Vesicle Concentration and Procoagulant Activity of Canine. **2019**, *60*, 423–429, doi:10.1111/jsap.13002.

24. Syrjä, P.; Palviainen, M.; Jokinen, T.; Kyöstilä, K.; Lohi, H.; Roosje, P.; Anderegg, L.; Leeb, T.; Sukura, A.; Eskelinen, E.L. Altered Basal Autophagy Affects Extracellular Vesicle Release in Cells of Lagotto Romagnolo Dogs With a Variant ATG4D. *Vet. Pathol.* **2020**, *57*, 926–935, doi:10.1177/0300985820959243.

25. Villatoro, A.J.; Alcoholado, C.; Martín-Astorga, M.C.; Fernández, V.; Cifuentes, M.; Becerra, J. Comparative Analysis and Characterization of Soluble Factors and Exosomes from Cultured Adipose Tissue and Bone Marrow Mesenchymal Stem Cells in Canine Species. *Vet. Immunol. Immunopathol.* **2019**, *208*, 6–15, doi:10.1016/j.vetimm.2018.12.003.

26. Villatoro, A.J.; Alcoholado, C.; Martín-Astorga, M.D.C.; Rico, G.; Fernández, V.; Becerra, J. Characterization of the Secretory Profile and Exosomes of Limbal Stem Cells in the Canine Species. *PLoS One* **2020**, *15*, e0244327, doi:10.1371/journal.pone.0244327.

27. Żmigrodzka, M.; Witkowska-Piłaszewicz, O.; Rzepecka, A.; Cywińska, A.; Jagielski, D.; Winnicka, A. Extracellular Vesicles in the Blood of Dogs with Cancer—a Preliminary Study. *Animals* **2019**, *9*, 575, doi:10.3390/ani9080575.

28. Tarique, I.; Haseeb, A.; Bai, X.; Li, W.; Yang, P.; Huang, Y.; Yang, S.; Xu, M.; Zhang, Y.; Vistro, W.A.; et al. Cellular Evidence of CD63-Enriched Exosomes and Multivesicular Bodies within the Seminiferous Tubule during the Spermatogenesis of Turtles. *Microsc. Microanal.* **2020**, *26*, 148–156, doi:10.1017/S1431927619015149.

29. Vistro, W.A.; Huang, Y.; Bai, X.; Yang, P.; Haseeb, A.; Chen, H.; Liu, Y.; Yue, Z.; Tarique, I.; Chen, Q. In Vivo Multivesicular Body and Exosome Secretion in the Intestinal Epithelial Cells of Turtles during Hibernation. *Microsc. Microanal.* **2019**, *25*, 1341–1351, doi:10.1017/S1431927619015071.

30. Waqas, M.Y.; Zhang, Q.; Ahmed, N.; Yang, P.; Xing, G.; Akhtar, M.; Basit, A.; Liu, T.; Hong, C.; Arshad, M.; et al. Cellular Evidence of Exosomes in the Reproductive Tract of Chinese Soft-Shelled Turtle Pelodiscus Sinensis. *J. Exp. Zool. Part A Ecol. Integr. Physiol.* **2017**, *327*, 18–27, doi:10.1002/jez.2065.

31. Almiñana, C.; Corbin, E.; Tsikis, G.; Alcântara-Neto, A.S.; Labas, V.; Reynaud, K.; Galio, L.; Uzbekov, R.; Garanina, A.S.; Druart, X.; et al. Oviduct Extracellular Vesicles Protein Content and Their Role during Oviduct–Embryo Cross-Talk. *Reproduction* **2017**, *154*, 253–268, doi:10.1530/REP-17-0054.

32. Banliat, C.; Le Bourhis, D.; Bernardi, O.; Tomas, D.; Labas, V.; Salvetti, P.; Guyonnet, B.; Mermillod, P.; Saint-Dizier, M. Oviduct Fluid Extracellular Vesicles Change the Phospholipid Composition of Bovine Embryos Developed in Vitro. *Int. J. Mol. Sci.* **2020**, *21*, 5326, doi:10.3390/ijms21155326.

33. Bauersachs, S.; Mermillod, P.; Almiñana, C. The Oviductal Extracellular Vesicles' RNA Cargo Regulates the Bovine Embryonic Transcriptome. *Int. J. Mol. Sci.* **2020**, *21*, 1303, doi:10.3390/ijms21041303.

34. Dissanayake, K.; Nõmm, M.; Lättekivi, F.; Ressaissi, Y.; Godakumara, K.; Lavrits, A.; Midekessa, G.; Viil, J.; Bæk, R.; Jørgensen, M.M.; et al. Individually Cultured Bovine Embryos Produce Extracellular Vesicles That Have the Potential to

Be Used as Non-Invasive Embryo Quality Markers. *Theriogenology* **2020**, *149*, 104–116, doi:10.1016/j.theriogenology.2020.03.008.

35. Bridi, A.; Andrade, G.M.; del Collado, M.; Sangalli, J.R.; de Ávila, A.C.F.C.M.; Motta, I.G.; da Silva, J.C.B.; Pugliesi, G.; Silva, L.A.; Meirelles, F. V.; et al. Small Extracellular Vesicles Derived from in Vivo- or in Vitro-Produced Bovine Blastocysts Have Different MiRNAs Profiles—Implications for Embryo-Maternal Recognition. *Mol. Reprod. Dev.* **2021**, *88*, 628–643, doi:10.1002/mrd.23527.

36. Kusama, K.; Nakamura, K.; Bai, R.; Nagaoka, K.; Sakurai, T.; Imakawa, K. Intrauterine Exosomes Are Required for Bovine Conceptus Implantation. *Biochem. Biophys. Res. Commun.* **2018**, *495*, 1370–1375, doi:10.1016/j.bbrc.2017.11.176.

37. Mazzarella, R.; Bastos, N.M.; Bridi, A.; del Collado, M.; Andrade, G.M.; Pinzon, J.; Prado, C.M.; Silva, L.A.; Meirelles, F.V.; Pugliesi, G.; et al. Changes in Oviductal Cells and Small Extracellular Vesicles MiRNAs in Pregnant Cows. *Front. Vet. Sci.* **2021**, *8*, 639752, doi:10.3389/fvets.2021.639752.

38. Nakamura, K.; Kusama, K.; Hori, M.; Imakawa, K. The Effect of Bta-MiR-26b in Intrauterine Extracellular Vesicles on Maternal Immune System during the Implantation Period. *Biochem. Biophys. Res. Commun.* **2021**, *573*, 100–106, doi:10.1016/j.bbrc.2021.08.019.

39. Pan, B.T.; Johnstone, R. Selective Externalization of the Transferrin Receptor by Sheep Reticulocytes in Vitro. Response to Ligands and Inhibitors of Endocytosis. *J. Biol. Chem.* **1984**, *259*, 9776–9782, doi:10.1016/s0021-9258(17)42767-0.

40. Pan, B.T.; Teng, K.; Wu, C.; Adam, M.; Johnstone, R.M. Electron Microscopic Evidence for Externalization of the Transferrin Receptor in Vesicular Form in Sheep Reticulocytes. *J. Cell Biol.* **1985**, *101*, 942–948, doi:10.1083/jcb.101.3.942.

41. Pioltine, E.M.; Machado, M.F.; da Silveira, J.C.; Fontes, P.K.; Botigelli, R.C.; Quaglio, A.E. V.; Costa, C.B.; Nogueira, M.F.G. Can Extracellular Vesicles from Bovine Ovarian Follicular Fluid Modulate the In-Vitro Oocyte Meiosis Progression Similarly to the CNP-NPR2 System? *Theriogenology* **2020**, *157*, 210–217, doi:10.1016/j.theriogenology.2020.06.031.

42. Qu, P.; Qing, S.; Liu, R.; Qin, H.; Wang, W.; Qiao, F.; Ge, H.; Liu, J.; Zhang, Y.; Cui, W.; et al. Effects of Embryo-Derived Exosomes on the Development of Bovine Cloned Embryos. *PLoS One* **2017**, *12*, e0174535, doi:10.1371/journal.pone.0174535.

43. da Silveira, J.C.; Andrade, G.M.; Simas, R.C.; Martins-Júnior, H.A.; Eberlin, M.N.; Smith, L.C.; Perecin, F.; Meirelles, F.V. Lipid Profile of Extracellular Vesicles and Their Relationship with Bovine Oocyte Developmental Competence: New Players in Intra Follicular Cell Communication. *Theriogenology* **2021**, *174*, 1–8, doi:10.1016/j.theriogenology.2021.07.024.

44. Sun, Y.; Xu, M.; Gao, R.; Xie, S.; Sun, X.; He, J.; Chen, X.; Li, Q.; Lu, S.; Yang, M.; et al. Identification of Differentially Expressed MiRNAs in Serum Extracellular Vesicles (EVs) of Kazakh Sheep at Early Pregnancy. *Reprod. Domest. Anim.* **2021**, *56*, 713–724, doi:10.1111/rda.13910.

45. Uzbekova, S.; Almiñana, C.; Labas, V.; Teixeira-Gomes, A.P.; Combes-Soia, L.; Tsikis, G.; Carvalho, A.V.; Uzbekov, R.; Singina, G. Protein Cargo of Extracellular Vesicles From Bovine Follicular Fluid and Analysis of Their Origin From Different Ovarian Cells. *Front. Vet. Sci.* **2020**, *7*, 584948, doi:10.3389/fvets.2020.584948.

46. Ying, W.; Hengqin, W.; Xiaomei, W.; Yunqi, Z.; Yong, Z.; Fusheng, Q. Extracellular Vesicles of Bovine Small Follicular Fluid Promote Ovarian Cortical Stromal Cell Proliferation and Steroidogenesis. *Reprod. Domest. Anim.* **2021**, *56*, 1425–1434, doi:10.1111/rda.14007.

47. Alcântara-Neto, A.S.; Schmaltz, L.; Caldas, E.; Blache, M.C.; Mermillod, P.; Almiñana, C. Porcine Oviductal Extracellular Vesicles Interact with Gametes and Regulate Sperm Motility and Survival. *Theriogenology* **2020**, *155*, 240–255, doi:10.1016/j.theriogenology.2020.05.043.

48. Barranco, I.; Padilla, L.; Parrilla, I.; Álvarez-Barrientos, A.; Pérez-Patiño, C.; Peña, F.J.; Martínez, E.A.; Rodriguez-Martínez, H.; Roca, J. Extracellular Vesicles Isolated from Porcine Seminal Plasma Exhibit Different Tetraspanin Expression Profiles. *Sci. Rep.* **2019**, *9*, 11584, doi:10.1038/s41598-019-48095-3.

49. Du, J.; Shen, J.; Wang, Y.; Pan, C.; Pang, W.; Diao, H.; Dong, W. Boar Seminal Plasma Exosomes Maintain Sperm Function by Infiltrating into the Sperm Membrane. *Oncotarget* **2016**, *7*, 58832–58847, doi:10.18632/oncotarget.11315.

50. Eirin, A.; Zhu, X.Y.; Puranik, A.S.; Woollard, J.R.; Tang, H.; Dasari, S.; Lerman, A.; Van Wijnen, A.J.; Lerman, L.O. Integrated Transcriptomic and Proteomic Analysis of the Molecular Cargo of Extracellular Vesicles Derived from Porcine Adipose Tissue-Derived Mesenchymal Stem Cells. *PLoS One* **2017**, *12*, e0174303,

doi:10.1371/journal.pone.0174303.

51. Luo, J.; Fan, Y.; Shen, L.; Niu, L.; Zhao, Y.; Jiang, D.; Zhu, L.; Jiang, A.; Tang, Q.; Ma, J.; et al. The Pro-Angiogenesis of Exosomes Derived from Umbilical Cord Blood of Intrauterine Growth Restriction Pigs Was Repressed Associated with MiRNAs. *Int. J. Biol. Sci.* **2018**, *14*, 1426–1436, doi:10.7150/ijbs.27029.

52. Pedrosa, A.C.; Andrade Torres, M.; Vilela Alkmin, D.; Pinzon, J.E.P.; Kitamura Martins, S.M.M.; Coelho da Silveira, J.; Furugen Cesar de Andrade, A. Spermatozoa and Seminal Plasma Small Extracellular Vesicles MiRNAs as Biomarkers of Boar Semen Cryotolerance. *Theriogenology* **2021**, *174*, 60–72, doi:10.1016/j.theriogenology.2021.07.022.

53. Zhang, J.; Luo, H.; Xiong, Z.; Wan, K.; Liao, Q.; He, H. High-Throughput Sequencing Reveals Biofluid Exosomal MiRNAs Associated with Immunity in Pigs. *Biosci. Biotechnol. Biochem.* **2020**, *84*, 53–62, doi:10.1080/09168451.2019.1661767.

54. Bowden, T.J.; Kraev, I.; Lange, S. Post-Translational Protein Deimination Signatures and Extracellular Vesicles (EVs) in the Atlantic Horseshoe Crab (Limulus Polyphemus). *Dev. Comp. Immunol.* **2020**, *110*, 103714, doi:10.1016/j.dci.2020.103714.

55. Bowden, T.J.; Kraev, I.; Lange, S. Extracellular Vesicles and Post-Translational Protein Deimination Signatures in Haemolymph of the American Lobster (Homarus Americanus). *Fish Shellfish Immunol.* **2020**, *106*, 79–102, doi:10.1016/j.fsi.2020.06.053.

56. Rast, J.P.; D'Alessio, S.; Kraev, I.; Lange, S. Post-Translational Protein Deimination Signatures in Sea Lamprey (Petromyzon Marinus) Plasma and Plasma-Extracellular Vesicles. *Dev. Comp. Immunol.* **2021**, *125*, 104225, doi:10.1016/j.dci.2021.104225.

57. Wang, M.; Liu, M.; Wang, B.; Jiang, K.; Jia, Z.; Wang, L.; Wang, L. Transcriptomic Analysis of Exosomal Shuttle MRNA in Pacific Oyster Crassostrea Gigas during Bacterial Stimulation. *Fish Shellfish Immunol.* **2018**, *74*, 540–550, doi:10.1016/j.fsi.2018.01.017.

58. Yang, H.; Li, X.; Ji, J.; Yuan, C.; Gao, X.; Zhang, Y.; Lu, C.; Li, F.; Zhang, X. Changes of MicroRNAs Expression Profiles from Red Swamp Crayfish (Procambarus Clarkia) Hemolymph Exosomes in Response to WSSV Infection. *Fish Shellfish Immunol.* **2019**, *84*, 169–177, doi:10.1016/j.fsi.2018.10.003.

59. Agrawal, A.K.; Aqil, F.; Jeyabalan, J.; Spencer, W.A.; Beck, J.; Gachuki, B.W.; Alhakeem, S.S.; Oben, K.; Munagala, R.; Bondada, S.; et al. Milk-Derived Exosomes for Oral Delivery of Paclitaxel. *Nanomedicine Nanotechnology, Biol. Med.* **2017**, *13*, 1627–1636, doi:10.1016/j.nano.2017.03.001.

60. Aguilar-Lozano, A.; Baier, S.; Grove, R.; Shu, J.; Giraud, D.; Leiferman, A.; Mercer, K.E.; Cui, J.; Badger, T.M.; Adamec, J.; et al. Concentrations of Purine Metabolites Are Elevated in Fluids from Adults and Infants and in Livers from Mice Fed Diets Depleted of Bovine Milk Exosomes and Their RNA Cargos. *J. Nutr.* **2018**, *148*, 1886–1894, doi:10.1093/jn/nxy223.

61. Aqil, F.; Munagala, R.; Jeyabalan, J.; Agrawal, A.K.; Kyakulaga, A.H.; Wilcher, S.A.; Gupta, R.C. Milk Exosomes - Natural Nanoparticles for SiRNA Delivery. *Cancer Lett.* **2019**, *449*, 186–195, doi:10.1016/j.canlet.2019.02.011.

62. Arntz, O.J.; Pieters, B.C.H.; Oliveira, M.C.; Broeren, M.G.A.; Bennink, M.B.; De Vries, M.; Van Lent, P.L.E.M.; Koenders, M.I.; Van den Berg, W.B.; Van der Kraan, P.M.; et al. Oral Administration of Bovine Milk Derived Extracellular Vesicles Attenuates Arthritis in Two Mouse Models. *Mol. Nutr. Food Res.* **2015**, *59*, 1701–1712, doi:10.1002/mnfr.201500222.

63. Badawy, A.A.; El-Magd, M.A.; AlSadrah, S.A. Therapeutic Effect of Camel Milk and Its Exosomes on MCF7 Cells In Vitro and In Vivo. *Integr. Cancer Ther.* **2018**, *17*, 1235–1246, doi:10.1177/1534735418786000.

64. Baier, S.R.; Nguyen, C.; Xie, F.; Wood, J.R.; Zempleni, J. MicroRNAs Are Absorbed in Biologically Meaningful Amounts from Nutritionally Relevant Doses of Cow Milk and Affect Gene Expression in Peripheral Blood Mononuclear Cells, HEK-293 Kidney Cell Cultures, and Mouse Livers. *J. Nutr.* **2014**, *144*, 1495–1500, doi:10.3945/jn.114.196436.

65. Benmoussa, A.; Ly, S.; Shan, S.T.; Laugier, J.; Boilard, E.; Gilbert, C.; Provost, P. A Subset of Extracellular Vesicles Carries the Bulk of MicroRNAs in Commercial Dairy Cow's Milk. *J. Extracell. Vesicles* **2017**, *6*, 1401897, doi:10.1080/20013078.2017.1401897.

66. Benmoussa, A.; Laugier, J.; Beauparlant, C.J.; Lambert, M.; Droit, A.; Provost, P. Complexity of the MicroRNA Transcriptome of Cow Milk and Milk-Derived Extracellular Vesicles Isolated via Differential Ultracentrifugation. *J. Dairy Sci.* **2020**, *103*, 16–29, doi:10.3168/jds.2019-16880.

67. Betker, J.L.; Angle, B.M.; Graner, M.W.; Anchordoquy, T.J. The Potential of Exosomes From Cow Milk for Oral Delivery. *J. Pharm. Sci.* **2019**, *108*, 1496–1505, doi:10.1016/j.xphs.2018.11.022.

68. Brown, B.A.; Zeng, X.; Todd, A.R.; Barnes, L.F.; Winstone, J.M.A.; Trinidad, J.C.; Novotny, M. V; Jarrold, M.F.; Clemmer, D.E. HHS Public Access. **2021**, *92*, 3285–3292, doi:10.1021/acs.analchem.9b05173.Charge.

69. Carobolante, G.; Mantaj, J.; Ferrari, E.; Vllasaliu, D. Cow Milk and Intestinal Epithelial Cell-Derived Extracellular Vesicles as Systems for Enhancing Oral Drug Delivery. *Pharmaceutics* **2020**, *12*, 226, doi:10.3390/pharmaceutics12030226.

70. Chen, T.; Xi, Q.Y.; Ye, R.S.; Cheng, X.; Qi, Q.E.; Wang, S.B.; Shu, G.; Wang, L.N.; Zhu, X.T.; Jiang, Q.Y.; et al. Exploration of MicroRNAs in Porcine Milk Exosomes. *BMC Genomics* **2014**, *15*, 100, doi:10.1186/1471-2164-15-100.

71. Xing, Y.; Cheng, Z.; Wang, R.; Lv, C.; James, T.D.; Yu, F. Analysis of Extracellular Vesicles as Emerging Theranostic Nanoplatforms. *Coord. Chem. Rev.* **2020**, *424*, 213506.

72. Chen, T.; Xie, M.Y.; Sun, J.J.; Ye, R.S.; Cheng, X.; Sun, R.P.; Wei, L.M.; Li, M.; Lin, D.L.; Jiang, Q.Y.; et al. Porcine Milk-Derived Exosomes Promote Proliferation of Intestinal Epithelial Cells. *Sci. Rep.* **2016**, *6*, 33862, doi:10.1038/srep33862.

73. Chen, T.; Xi, Q.Y.; Sun, J.J.; Ye, R.S.; Cheng, X.; Sun, R.P.; Wang, S.B.; Shu, G.; Wang, L.N.; Zhu, X.T.; et al. Revelation of MRNAs and Proteins in Porcine Milk Exosomes by Transcriptomic and Proteomic Analysis. *BMC Vet. Res.* **2017**, *13*, 101, doi:10.1186/s12917-017-1021-8.

74. Chen, W.; Wang, R.; Li, D.; Zuo, C.; Wen, P.; Liu, H.; Chen, Y.; Fujita, M.; Wu, Z.; Yang, G. Comprehensive Analysis of the Glycome and Glycoproteome of Bovine Milk-Derived Exosomes. *J. Agric. Food Chem.* **2020**, *68*, 12692-12701, doi:10.1021/acs.jafc.0c04605.

75. Chen, Z.; Xie, Y.; Luo, J.; Chen, T.; Xi, Q.; Zhang, Y.; Sun, J. Milk Exosome-Derived MiRNAs from Water Buffalo Are Implicated in Immune Response and Metabolism Process. *BMC Vet. Res.* **2020**, *16*, 123, doi:10.1186/s12917-020-02339-x.

76. Colitti, M.; Sgorlon, S.; Licastro, D.; Stefanon, B. Differential Expression of MiRNAs in Milk Exosomes of Cows Subjected to Group Relocation. *Res. Vet. Sci.* **2019**, *122*, 148–155, doi:10.1016/j.rvsc.2018.11.024.

77. Del Pozo-Acebo, L.; de las Hazas, M.C.L.; Tomé-Carneiro, J.; Gil-Cabrerizo, P.; San-Cristobal, R.; Busto, R.; García-Ruiz, A.; Dávalos, A. Bovine Milk-Derived Exosomes as a Drug Delivery Vehicle for Mirna-Based Therapy. *Int. J. Mol. Sci.* **2021**, *22*, 1105, doi:10.3390/ijms22031105.

78. Ferreira, R.F.; Blees, T.; Shakeri, F.; Buness, A.; Sylvester, M.; Savoini, G.; Agazzi, A.; Mrljak, V.; Sauerwein, H. Comparative Proteome Profiling in Exosomes Derived from Porcine Colostrum versus Mature Milk Reveals Distinct Functional Proteomes. *J. Proteomics* **2021**, *249*, 104338, doi:10.1016/j.jprot.2021.104338.

79. Gao, H.N.; Guo, H.Y.; Zhang, H.; Xie, X.L.; Wen, P.C.; Ren, F.Z. Yak-Milk-Derived Exosomes Promote Proliferation of Intestinal Epithelial Cells in an Hypoxic Environment. *J. Dairy Sci.* **2019**, *102*, 985–996, doi:10.3168/jds.2018-14946.

80. Golan-Gerstl, R.; Elbaum Shiff, Y.; Moshayoff, V.; Schecter, D.; Leshkowitz, D.; Reif, S. Characterization and Biological Function of Milk-Derived MiRNAs. *Mol. Nutr. Food Res.* **2017**, *61*, doi:10.1002/mnfr.201700009.

81. Grossen, P.; Portmann, M.; Koller, E.; Duschmalé, M.; Minz, T.; Sewing, S.; Pandya, N.J.; van Geijtenbeek, S.K.; Ducret, A.; Kusznir, E.A.; et al. Evaluation of Bovine Milk Extracellular Vesicles for the Delivery of Locked Nucleic Acid Antisense Oligonucleotides. *Eur. J. Pharm. Biopharm.* **2021**, *158*, 198–210, doi:10.1016/j.ejpb.2020.11.012.

82. Gu, Y.; Li, M.; Wang, T.; Liang, Y.; Zhong, Z.; Wang, X.; Zhou, Q.; Chen, L.; Lang, Q.; He, Z.; et al. Lactation-Related MicroRNA Expression Profiles of Porcine Breast Milk Exosomes. *PLoS One* **2012**, *7*, e43691, doi:10.1371/journal.pone.0043691.

83. Hansen, M.S.; Gadegaard, I.S.E.; Arnspang, E.C.; Blans, K.; Nejsum, L.N.; Rasmussen, J.T. Specific and Non-Invasive Fluorescent Labelling of Extracellular Vesicles for Evaluation of Intracellular Processing by Intestinal Epithelial Cells. *Biomedicines* **2020**, *8*, 211, doi:10.3390/BIOMEDICINES8070211.

84. Hata, T.; Murakami, K.; Nakatani, H.; Yamamoto, Y.; Matsuda, T.; Aoki, N. Isolation of Bovine Milk-Derived Microvesicles Carrying MRNAs and MicroRNAs. *Biochem. Biophys. Res. Commun.* **2010**, *396*, 528–533, doi:10.1016/j.bbrc.2010.04.135.

85. Herwijnen, M.J.C. va.; Driedonks, T.A.P.; Snoek, B.L.; Kroon, A.M.T.; Kleinjan, M.; Jorritsma, R.; Pieterse, C.M.J.; Hoen, E.N.M.N.; Wauben, M.H.M. Abundantly Present MiRNAs in Milk-Derived Extracellular Vesicles Are Conserved Between Mammals. *Front. Nutr.* **2018**, *5*, 81, doi:10.3389/fnut.2018.00081.

86. Ibrahim, H.M.; Mohammed-Geba, K.; Tawfic, A.A.; El-Magd, M.A. Camel Milk Exosomes Modulate Cyclophosphamide-Induced Oxidative Stress and Immuno-Toxicity in Rats. *Food Funct.* **2019**, *10*, 7523–7532,

doi:10.1039/c9fo01914f.

87. Izumi, H.; Tsuda, M.; Sato, Y.; Kosaka, N.; Ochiya, T.; Iwamoto, H.; Namba, K.; Takeda, Y. Bovine Milk Exosomes Contain MicroRNA and MRNA and Are Taken up by Human Macrophages. *J. Dairy Sci.* **2015**, *98*, 2920–2933, doi:10.3168/jds.2014-9076.

88. Kirchner, B.; Buschmann, D.; Paul, V.; Pfaffl, M.W. Postprandial Transfer of Colostral Extracellular Vesicles and Their Protein and MiRNA Cargo in Neonatal Calves. *PLoS One* **2020**, *15*, e0229606, doi:10.1371/journal.pone.0229606.

89. Komine-Aizawa, S.; Ito, S.; Aizawa, S.; Namiki, T.; Hayakawa, S. Cow Milk Exosomes Activate NK Cells and ΓδT Cells in Human PBMCs in Vitro. *Immunol. Med.* **2020**, *43*, 161–170, doi:10.1080/25785826.2020.1791400.

90. Lee, B.H.; Chen, B.R.; Huang, C.T.; Lin, C.H. The Immune Activity of Pt-Peptide Derived from Anti-Lipopolysaccharide Factor of the Swimming Crab Portunus Trituberculatus Is Enhanced When Encapsulated in Milk-Derived Extracellular Vesicles. *Mar. Drugs* **2019**, *17*, 248, doi:10.3390/md17050248.

91. Leiferman, A.; Shu, J.; Grove, R.; Cui, J.; Adamec, J.; Zempleni, J. A Diet Defined by Its Content of Bovine Milk Exosomes and Their RNA Cargos Has Moderate Effects on Gene Expression, Amino Acid Profiles and Grip Strenght in Skeletal Muscle in C57BL/l Mice. *J nutr Biochem* **2016**, *59*, 123–128, doi:10.1016/j.jnutbio.2018.06.007.A.

92. Lin, D.; Chen, T.; Xie, M.; Li, M.; Zeng, B.; Sun, R.; Zhu, Y.; Ye, D.; Wu, J.; Sun, J.; et al. Oral Administration of Bovine and Porcine Milk Exosome Alter MiRNAs Profile in Piglet Serum. *Sci. Rep.* **2020**, *10*, 6983, doi:10.1016/j.canlet.2017.02.004.

93. Luo, S.; Sun, X.; Huang, M.; Ma, Q.; Du, L.; Cui, Y. Enhanced Neuroprotective Effects of Epicatechin Gallate Encapsulated by Bovine Milk-Derived Exosomes against Parkinson's Disease through Antiapoptosis and Antimitophagy. *J. Agric. Food Chem.* **2021**, *69*, 5134–5143, doi:10.1021/acs.jafc.0c07658.

94. Ma, J.; Wang, C.; Long, K.; Zhang, H.; Zhang, J.; Jin, L.; Tang, Q.; Jiang, A.; Wang, X.; Tian, S.; et al. Exosomal MicroRNAs in Giant Panda (Ailuropoda Melanoleuca) Breast Milk: Potential Maternal Regulators for the Development of Newborn Cubs. *Sci. Rep.* **2017**, *7*, 3507, doi:10.1038/s41598-017-03707-8.

95. Ma, S.; Tong, C.; Ibeagha-Awemu, E.M.; Zhao, X. Identification and Characterization of Differentially Expressed Exosomal MicroRNAs in Bovine Milk Infected with Staphylococcus Aureus. *BMC Genomics* **2019**, *20*, 934, doi:10.1186/s12864-019-6338-1.

96. Manca, S.; Upadhyaya, B.; Mutai, E.; Desaulniers, A.T.; Cederberg, R.A.; White, B.R.; Zempleni, J. Milk Exosomes Are Bioavailable and Distinct MicroRNA Cargos Have Unique Tissue Distribution Patterns. *Sci. Rep.* **2018**, *8*, 11321, doi:10.1038/s41598-018-29780-1.

97. Marsh, S.R.; Pridham, K.J.; Jourdan, J.; Gourdie, R.G. Novel Protocols for Scalable Production of High Quality Purified Small Extracellular Vesicles from Bovine Milk. *Nanotheranostics* **2021**, *5*, 488–498, doi:10.7150/ntno.62213.

98. Mecocci, S.; Gevi, F.; Pietrucci, D.; Cavinato, L.; Luly, F.R.; Pascucci, L.; Petrini, S.; Ascenzioni, F.; Zolla, L.; Chillemi, G.; et al. Anti-Inflammatory Potential of Cow, Donkey and Goat Milk Extracellular Vesicles as Revealed by Metabolomic Profile. *Nutrients* **2020**, *12*, 2908, doi:10.3390/nu12102908.

99. Mellisho, E.A.; Briones, M.A.; Velásquez, A.E.; Cabezas, J.; Castro, F.O.; Rodríguez-Álvarez, L. Extracellular Vesicles Secreted during Blastulation Show Viability of Bovine Embryos. *Reproduction* **2019**, *158*, 477–192.

100. Meng, Y.; Eirin, A.; Zhu, X.Y.; O'Brien, D.R.; Lerman, A.; Van Wijnen, A.J.; Lerman, L.O. The Metabolic Syndrome Modifies the MRNA Expression Profile of Extracellular Vesicles Derived from Porcine Mesenchymal Stem Cells. *Diabetol. Metab. Syndr.* **2018**, *10*, 58.

101. Modepalli, V.; Kumar, A.; Hinds, L.A.; Sharp, J.A.; Nicholas, K.R.; Lefevre, C. Differential Temporal Expression of Milk MiRNA during the Lactation Cycle of the Marsupial Tammar Wallaby (Macropus Eugenii). *BMC Genomics* **2014**, *15*, 1012, doi:10.1186/1471-2164-15-1012.

102. Munga; Aqil, F.; Jeyabalan, J.; Agrawal, A.K.; Mudd, A.M.; Kyakulaga, A.H.; Singh, I.P.; Vadhanam, M. V.; Gupta, R.C. Exosomal Formulation of Anthocyanidins against Multiple Cancer Types. *Cancer Lett.* **2017**, *393*, 94–102, doi:10.1016/j.canlet.2017.02.004.Exosomal.

103. Özdemir, S. Identification and Comparison of Exosomal MicroRNAs in the Milk and Colostrum of Two Different Cow Breeds. *Gene* **2020**, *743*, 144609, doi:10.1016/j.gene.2020.144609.

104. Parry, H.A.; Brooks Mobley, C.; Mumford, P.W.; Romero, M.A.; Haun, C.T.; Zhang, Y.; Roberson, P.A.; Zempleni, J.;

Ferrando, A.A.; Vechetti, I.J.; et al. Bovine Milk Extracellular Vesicles (EVs) Modification Elicits Skeletal Muscle Growth in Rats. *Front. Physiol.* **2019**, *10*, 436, doi:10.3389/fphys.2019.00436.

105. Pieters, B.C.H.; J.Arntz, O.; Bennink, M.B.; Broeren, M.G.A.; Van Caam, A.P.M.; Koenders, M.I.; Van Lent, P.L.E.M.; Van Den Berg, W.B.; De Vries, M.; Van Der Kraan, P.M.; et al. Commercial Cow Milk Contains Physically Stable Extracellular Vesicles Expressing Immunoregulatory TGF-β. *PLoS One* **2015**, *10*, e0121123, doi:10.1371/journal.pone.0121123.

106. Reif, S.; Elbaum-Shiff, Y.; Koroukhov, N.; Shilo, I.; Musseri, M.; Golan-Gerstl, R. Cow and Human Milk-Derived Exosomes Ameliorate Colitis in Dss Murine Model. *Nutrients* **2020**, *12*, 2589, doi:10.3390/nu12092589.

107. Reinhardt, T.A.; Lippolis, J.D.; Nonnecke, B.J.; Sacco, R.E. Bovine Milk Exosome Proteome. *J. Proteomics* **2012**, *75*, 1486–1492, doi:10.1016/j.jprot.2011.11.017.

108. Reinhardt, T.A.; Sacco, R.E.; Nonnecke, B.J.; Lippolis, J.D. Bovine Milk Proteome: Quantitative Changes in Normal Milk Exosomes, Milk Fat Globule Membranes and Whey Proteomes Resulting from Staphylococcus Aureus Mastitis. *J. Proteomics* **2013**, *82*, 141–154, doi:10.1016/j.jprot.2013.02.013.

109. Ross, M.; Atalla, H.; Karrow, N.; Mallard, B.A. The Bioactivity of Colostrum and Milk Exosomes of High, Average, and Low Immune Responder Cows on Human Intestinal Epithelial Cells. *J. Dairy Sci.* **2021**, *104*, 2499–2510, doi:10.3168/jds.2020-18405.

110. Samuel, M.; Chisanga, D.; Liem, M.; Keerthikumar, S.; Anand, S.; Ang, C.S.; Adda, C.G.; Versteegen, E.; Jois, M.; Mathivanan, S. Bovine Milk-Derived Exosomes from Colostrum Are Enriched with Proteins Implicated in Immune Response and Growth. *Sci. Rep.* **2017**, *7*, 5933, doi:10.1038/s41598-017-06288-8.

111. Santos, G.; Bottino, M.P.; Santos, A.P.C.; Simões, L.M.S.; Souza, J.C.; Ferreira, M.B.D.; da Silveira, J.C.; Ávila, A.C.F.C.M.; Bride, A.; Sales, J.N.S. Subclinical Mastitis Interferes with Ovulation, Oocyte and Granulosa Cell Quality in Dairy Cows. *Theriogenology* **2018**, *119*, 214–219, doi:10.1016/j.theriogenology.2018.04.028.

112. Sedykh, S.E.; Purvinish, L. V.; Monogarov, A.S.; Burkova, E.E.; Grigor'eva, A.E.; Bulgakov, D. V.; Dmitrenok, P.S.; Vlassov, V. V.; Ryabchikova, E.I.; Nevinsky, G.A. Purified Horse Milk Exosomes Contain an Unpredictable Small Number of Major Proteins. *Biochim. Open* **2017**, *4*, 61–72, doi:10.1016/j.biopen.2017.02.004.

113. Stremmel, W.; Weiskirchen, R.; Melnik, B.C. Milk Exosomes Prevent Intestinal Inflammation in a Genetic Mouse Model of Ulcerative Colitis: A Pilot Experiment. *Inflamm. Intest. Dis.* **2020**, *5*, 117–123, doi:10.1159/000507626.

114. Sun, J.; Aswath, K.; Schroeder, S.G.; Lippolis, J.D.; Reinhardt, T.A.; Sonstegard, T.S. MicroRNA Expression Profiles of Bovine Milk Exosomes in Response to Staphylococcus Aureus Infection. *BMC Genomics* **2015**, *16*, 806, doi:10.1186/s12864-015-2044-9.

115. Sun, Y.; Wang, C.; Sun, X.; Guo, M. Comparative Proteomics of Whey and Milk Fat Globule Membrane Proteins of Guanzhong Goat and Holstein Cow Mature Milk. *J. Food Sci.* **2019**, *84*, 244–253, doi:10.1111/1750-3841.14428.

116. Sun, Y.; Wang, C.; Sun, X.; Jiang, S.; Guo, M. Characterization of the Milk Fat Globule Membrane Proteome in Colostrum and Mature Milk of Xinong Saanen Goats. *J. Dairy Sci.* **2020**, *103*, 3017–3024, doi:10.3168/jds.2019-17739.

117. Sun, J.; Xiong, J.; Yao, L.; Chen, T.; Luo, J.; Xi, Q.; Zhang, Y. The Effect of Dietary Ginseng Polysaccharide Supplementation on Porcine Milk-Derived EsRNAs Involved in the Host Immune Responses. *J. Anim. Physiol. Anim. Nutr. (Berl).* **2019**, *103*, 276–282, doi:10.1111/jpn.12993.

118. Tao, H.; Xu, H.; Zuo, L.; Li, C.; Qiao, G.; Guo, M.; Zheng, L.; Leitgeb, M.; Lin, X. Exosomes-Coated Bcl-2 SiRNA Inhibits the Growth of Digestive System Tumors Both in Vitro and in Vivo. *Int. J. Biol. Macromol.* **2020**, *161*, 470–480, doi:10.1016/j.ijbiomac.2020.06.052.

119. Nordgren, T.M.; Heires, A.J.; Zempleni, J.; Swanson, B.J.; Wichman, C.; Romberger, D.J. Bovine Milk-Derived Extracellular Vesicles Enhance Inflammation and Promote M1 Polarization Following Agricultural Dust Exposure in Mice. *J. Nutr. Biochem.* **2019**, *64*, 110–120, doi:10.1016/j.jnutbio.2018.10.017.

120. Villatoro, A.J.; Martín-Astorga, M. del C.; Alcoholado, C.; Becerra, J. Canine Colostrum Exosomes: Characterization and Influence on the Canine Mesenchymal Stem Cell Secretory Profile and Fibroblast Anti-Oxidative Capacity. *BMC Vet. Res.* **2020**, *16*, 417, doi:10.1186/s12917-020-02623-w.

121. Wang, L.; Wang, X.; Shi, Z.; Shen, L.; Zhang, J.; Zhang, J. Bovine Milk Exosomes Attenuate the Alteration of Purine Metabolism and Energy Status in IEC-6 Cells Induced by Hydrogen Peroxide. *Food Chem.* **2021**, *350*, 129142, doi:10.1016/j.foodchem.2021.129142.

122. Wang, L.; Shi, Z.; Wang, X.; Mu, S.; Xu, X.; Shen, L.; Ping, L. Protective Effects of Bovine Milk Exosomes against Oxidative Stress in IEC-6 Cells. *Eur. J. Nutr.* **2021**, *60*, 317–327.

123. Wolf, T.; Baier, S.R.; Zempleni, J. The Intestinal Transport of Bovine Milk Exosomes Is Mediated by Endocytosis in Human Colon Carcinoma Caco-2 Cells and Rat Small Intestinal IEC-6 Cells. *J Nutr* **2015**, *10*, 2201–2206.

124. Xie, M.Y.; Hou, L.J.; Sun, J.J.; Zeng, B.; Xi, Q.Y.; Luo, J.Y.; Chen, T.; Zhang, Y.L. Porcine Milk Exosome MiRNAs Attenuate LPS-Induced Apoptosis through Inhibiting TLR4/NF-KB and P53 Pathways in Intestinal Epithelial Cells. *J. Agric. Food Chem.* **2019**, *67*, 9477–9491, doi:10.1021/acs.jafc.9b02925.

125. Yang, M.; Song, D.; Cao, X.; Wu, R.; Liu, B.; Ye, W.; Wu, J.; Yue, X. Comparative Proteomic Analysis of Milk-Derived Exosomes in Human and Bovine Colostrum and Mature Milk Samples by ITRAQ-Coupled LC-MS/MS. *Food Res. Int.* **2017**, *92*, 17–25, doi:10.1016/j.foodres.2016.11.041.

126. Zeng, B.; Chen, T.; Xie, M.Y.; Luo, J.Y.; He, J.J.; Xi, Q.Y.; Sun, J.J.; Zhang, Y.L. Exploration of Long Noncoding RNA in Bovine Milk Exosomes and Their Stability during Digestion in Vitro. *J. Dairy Sci.* **2019**, *102*, 6726–6737, doi:10.3168/jds.2019-16257.

127. Zeng, B.; Wang, H.; Luo, J.; Xie, M.; Zhao, Z.; Chen, X.; Wang, D.; Sun, J.; Xi, Q.; Chen, T.; et al. Porcine Milk-Derived Small Extracellular Vesicles Promote Intestinal Immunoglobulin Production through Pigr. *Animals* **2021**, *11*, 1522, doi:10.3390/ani11061522.

128. Zhang, M.; Ma, Z.; Li, R.; Guo, S.; Qiu, Y.; Gao, X. Proteomic Analysis Reveals Proteins and Pathways Associated with Lactation in Bovine Mammary Epithelial Cell-Derived Exosomes. *J. Proteome Res.* **2020**, *19*, 3211–3219, doi:10.1021/acs.jproteome.0c00176.

129. Zhou, F.; Paz, H.A.; Sadri, M.; Cui, J.; Kachman, S.D.; Fernando, S.C.; Zempleni, J. Dietary Bovine Milk Exosomes Elicit Changes in Bacterial Communities in C57BL/6 Mice. *Am. J. Physiol. Gastrointest. Liver Physiol.* **2019**, *317*, G618–G624.

130. Abeysinghe, P.; Turner, N.; Garcia, I.M.; Mosaad, E.; Peiris, H.N.; Mitchell, M.D. The Role of Exosomal Epigenetic Modifiers in Cell Communication and Fertility of Dairy Cows. *Int. J. Mol. Sci.* **2020**, *21*, 9106, doi:10.3390/ijms21239106.

131. Colitti, M.; Sgorlon, S.; Stefanon, B. Exosome Cargo in Milk as a Potential Marker of Cow Health. *J. Dairy Res.* **2020**, *87*, 79–83, doi:10.1017/S0022029920000485.

132. Gigli, I.; Maizon, D.O. MicroRNAs and the Mammary Gland: A New Understanding of Gene Expression. *Genet. Mol. Biol.* **2013**, *36*, 465–474, doi:10.1590/S1415-47572013005000040.

133. Maity, S.; Bhat, A.H.; Giri, K.; Ambatipudi, K. BoMiProt: A Database of Bovine Milk Proteins. *J. Proteomics* **2020**, *215*, 103648, doi:10.1016/j.jprot.2020.103648.

134. Melnik, B.C.; John, S.M.; Schmitz, G. Milk: An Exosomal MicroRNA Transmitter Promoting Thymic Regulatory T Cell Maturation Preventing the Development of Atopy? *J. Transl. Med.* **2014**, *12*, 43, doi:10.1186/1479-5876-12-43.

135. Melnik, B.C.; Schmitz, G. MicroRNAs: Milk's Epigenetic Regulators. *Best Pract. Res. Clin. Endocrinol. Metab.* **2017**, *31*, 427–442, doi:10.1016/j.beem.2017.10.003.

136. Melnik, B.C.; Schmitz, G. Exosomes of Pasteurized Milk: Potential Pathogens of Western Diseases. *J. Transl. Med.* **2019**, *17*, 3, doi:10.1186/s12967-018-1760-8.

137. Melnik, B.C. Lifetime Impact of Cow's Milk on Overactivation of Mtorc1: From Fetal to Childhood Overgrowth, Acne, Diabetes, Cancers, and Neurodegeneration. *Biomolecules* **2021**, *11*, 404, doi:10.3390/biom11030404.

138. Melnik, B.C.; Schmitz, G. Pasteurized Non-Fermented Cow's Milk but Not Fermented Milk Is a Promoter of MTORC1-Driven Aging and Increased Mortality. *Ageing Res. Rev.* **2021**, *67*, 101270, doi:10.1016/j.arr.2021.101270.

139. Ortega-Anaya, J.; Jiménez-Flores, R. Symposium Review: The Relevance of Bovine Milk Phospholipids in Human Nutrition—Evidence of the Effect on Infant Gut and Brain Development. *J. Dairy Sci.* **2019**, *102*, 2738–2748, doi:10.3168/jds.2018-15342.

140. Wehbe, Z.; Kreydiyyeh, S. Cow's Milk May Be Delivering Potentially Harmful Undetected Cargoes to Humans. Is It Time to Reconsider Dairy Recommendations? *Nutr. Rev.* **2022**, *80*, 874–888, doi:10.1093/nutrit/nuab046.

141. Zhang, S.; Chen, F.; Zhang, Y.; Lv, Y.; Heng, J.; Min, T.; Li, L.; Guan, W. Recent Progress of Porcine Milk Components and Mammary Gland Function. *J. Anim. Sci. Biotechnol.* **2018**, *9*, 77, doi:10.1186/s40104-018-0291-8.
